# Whole transcriptome profiling of liquid biopsies from tumour xenografted mouse models enables specific monitoring of tumour-derived extracellular RNA

**DOI:** 10.1093/narcan/zcac037

**Published:** 2022-11-28

**Authors:** Vanessa Vermeirssen, Jill Deleu, Annelien Morlion, Celine Everaert, Jilke De Wilde, Jasper Anckaert, Kaat Durinck, Justine Nuytens, Muhammad Rishfi, Frank Speleman, Hanne Van Droogenbroeck, Kimberly Verniers, Maria Francesca Baietti, Maarten Albersen, Eleonora Leucci, Edward Post, Myron G Best, Tom Van Maerken, Bram De Wilde, Jo Vandesompele, Anneleen Decock

**Affiliations:** Lab for Computational Biology, Integromics and Gene Regulation (CBIGR), Cancer Research Institute Ghent (CRIG), 9000, Ghent, Belgium; Department of Biomedical Molecular Biology, Ghent University, 9000, Ghent, Belgium; OncoRNALab, Cancer Research Institute Ghent (CRIG), 9000, Ghent, Belgium; Department of Biomolecular Medicine, Ghent University, 9000, Ghent, Belgium; OncoRNALab, Cancer Research Institute Ghent (CRIG), 9000, Ghent, Belgium; Department of Biomolecular Medicine, Ghent University, 9000, Ghent, Belgium; OncoRNALab, Cancer Research Institute Ghent (CRIG), 9000, Ghent, Belgium; Department of Biomolecular Medicine, Ghent University, 9000, Ghent, Belgium; OncoRNALab, Cancer Research Institute Ghent (CRIG), 9000, Ghent, Belgium; Department of Biomolecular Medicine, Ghent University, 9000, Ghent, Belgium; Department of Biomolecular Medicine, Ghent University, 9000, Ghent, Belgium; Department of Pathology, Ghent University Hospital, 9000, Ghent, Belgium; OncoRNALab, Cancer Research Institute Ghent (CRIG), 9000, Ghent, Belgium; Department of Biomolecular Medicine, Ghent University, 9000, Ghent, Belgium; Department of Biomolecular Medicine, Ghent University, 9000, Ghent, Belgium; Pediatric Precision Oncology Lab (PPOL), Cancer Research Institute Ghent (CRIG), 9000, Ghent, Belgium; OncoRNALab, Cancer Research Institute Ghent (CRIG), 9000, Ghent, Belgium; Department of Biomolecular Medicine, Ghent University, 9000, Ghent, Belgium; Department of Biomolecular Medicine, Ghent University, 9000, Ghent, Belgium; Pediatric Precision Oncology Lab (PPOL), Cancer Research Institute Ghent (CRIG), 9000, Ghent, Belgium; Department of Biomolecular Medicine, Ghent University, 9000, Ghent, Belgium; Pediatric Precision Oncology Lab (PPOL), Cancer Research Institute Ghent (CRIG), 9000, Ghent, Belgium; OncoRNALab, Cancer Research Institute Ghent (CRIG), 9000, Ghent, Belgium; Department of Biomolecular Medicine, Ghent University, 9000, Ghent, Belgium; OncoRNALab, Cancer Research Institute Ghent (CRIG), 9000, Ghent, Belgium; Department of Biomolecular Medicine, Ghent University, 9000, Ghent, Belgium; Laboratory for RNA Cancer Biology, Department of Oncology, KU Leuven, 3000, Leuven, Belgium; TRACE, Leuven Cancer Institute, KU Leuven, 3000, Leuven, Belgium; Department of Development and Regeneration, Laboratory of Experimental Urology, KU Leuven, Department of Urology, University Hospitals Leuven, 3000, Leuven, Belgium; Laboratory for RNA Cancer Biology, Department of Oncology, KU Leuven, 3000, Leuven, Belgium; TRACE, Leuven Cancer Institute, KU Leuven, 3000, Leuven, Belgium; Amsterdam UMC Location Vrije Universiteit Amsterdam, Department of Neurosurgery, Boelelaan 1117, 1081 HV, Amsterdam, the Netherlands; Cancer Center Amsterdam, Brain Tumor Center and Liquid Biopsy Center, 1081 HV, Amsterdam, the Netherlands; Amsterdam UMC Location Vrije Universiteit Amsterdam, Department of Neurosurgery, Boelelaan 1117, 1081 HV, Amsterdam, the Netherlands; Cancer Center Amsterdam, Brain Tumor Center and Liquid Biopsy Center, 1081 HV, Amsterdam, the Netherlands; OncoRNALab, Cancer Research Institute Ghent (CRIG), 9000, Ghent, Belgium; Department of Biomolecular Medicine, Ghent University, 9000, Ghent, Belgium; Department of Laboratory Medicine, AZ Groeninge, 8500, Kortrijk, Belgium; OncoRNALab, Cancer Research Institute Ghent (CRIG), 9000, Ghent, Belgium; Department of Biomolecular Medicine, Ghent University, 9000, Ghent, Belgium; Department of Paediatric Haematology Oncology and Stem Cell Transplantation, Ghent University Hospital, 9000, Ghent, Belgium; OncoRNALab, Cancer Research Institute Ghent (CRIG), 9000, Ghent, Belgium; Department of Biomolecular Medicine, Ghent University, 9000, Ghent, Belgium; OncoRNALab, Cancer Research Institute Ghent (CRIG), 9000, Ghent, Belgium; Department of Biomolecular Medicine, Ghent University, 9000, Ghent, Belgium

## Abstract

While cell-free DNA (cfDNA) is widely being investigated, free circulating RNA (extracellular RNA, exRNA) has the potential to improve cancer therapy response monitoring and detection due to its dynamic nature. However, it remains unclear in which blood subcompartment tumour-derived exRNAs primarily reside. We developed a host-xenograft deconvolution framework, exRNAxeno, with mapping strategies to either a combined human-mouse reference genome or both species genomes in parallel, applicable to exRNA sequencing data from liquid biopsies of human xenograft mouse models. The tool enables to distinguish (human) tumoural RNA from (murine) host RNA, to specifically analyse tumour-derived exRNA. We applied the combined pipeline to total exRNA sequencing data from 95 blood-derived liquid biopsy samples from 30 mice, xenografted with 11 different tumours. Tumoural exRNA concentrations are not determined by plasma platelet levels, while host exRNA concentrations increase with platelet content. Furthermore, a large variability in exRNA abundance and transcript content across individual mice is observed. The tumoural gene detectability in plasma is largely correlated with the RNA expression levels in the tumour tissue or cell line. These findings unravel new aspects of tumour-derived exRNA biology in xenograft models and open new avenues to further investigate the role of exRNA in cancer.

## Graphical Abstract

**Graphical Abstract:**
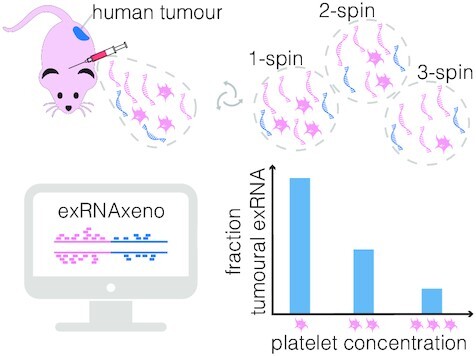
Upon whole transcriptome profiling of plasma from xenografted mice, the computational framework exRNAxeno reliably quantifies tumoural extracellular RNA (exRNA) and increasing platelet concentrations reveal lower fractions of tumoural exRNA.

## INTRODUCTION

Tumour cells both actively and passively release their content into the blood stream (1). This has opened unprecedented opportunities for accessing tumour-derived molecules without the need to perform an invasive biopsy or surgery aiding in diagnosis and therapy response monitoring. To this end, only a small volume of blood is required. These blood-based liquid biopsies are minimally invasive and therefore more readily executable than tissue biopsies in certain circumstances. They may also provide a more comprehensive picture of the genomic make-up of the entire cancer as liquid biopsies are not restricted to a specific local tumour sampling site. A major advantage is the possibility towards longitudinal sampling, which is in most cases impossible for solid tumours, thus facilitating treatment response monitoring in patients (2). Taken together, these advantages predict an important role for blood-based liquid biopsies in the emerging era of precision oncology (3). Different types of biomolecules within liquid biopsies are being evaluated, including free circulating DNA and RNA. While cell-free DNA (cfDNA) as a blood-based biosource is widely being used and investigated, clinical applications of extracellular RNA (exRNA) are scarce (3,4). Nevertheless, it was recently shown that exRNAs are present in a wide diversity of (blood-based) liquid biopsies and can act as promising precision oncology biomarkers (5–8).

A fundamental open question hampering the set-up of large-scale liquid biopsy collections for tumour exRNA analysis is that it remains unclear in which blood subcompartment tumour-derived exRNAs primarily reside. Blood plasma is amongst the most studied liquid biopsies, but recently also tumour-educated platelets (TEPs) have emerged as a promising liquid biopsy. TEPs arise by exposing blood platelets to tumour cells and to external signals in the tumour microenvironment, leading to the transfer and storage of tumour-associated biomolecules and to the induction of protein translation and specific RNA splicing events in the platelets. Moreover, the RNA content and RNA splicing patterns of these TEPs are claimed to be cancer-specific, therefore serving as cancer biomarkers (9–17). Consequently, differences in plasma preparation procedures, resulting in different amounts of blood platelets being present in samples, may influence the tumour signal that is detected in plasma. It was previously shown that different plasma fractions from cancer patients indeed show distinct exRNA profiles and that particular tumour-specific mutations can only be detected in certain blood-fractions. However, in-depth transcriptome-wide studies specifically focusing on tumour-derived exRNA in different cell-free blood fractions are completely lacking (18,19).

Although tumour-specific structural aberrations, such as fusion genes and single nucleotide or insertion/deletion mutations can be detected in the exRNA of cancer patients, charting the entire tumour-derived extracellular transcriptome is impossible, as tumoural exRNA generally cannot be distinguished from non-tumoural exRNA in the patient's blood (20–23). Therefore, to characterize a tumour’s extracellular transcriptome by massively parallel sequencing, human xenograft models are excellent tools, as tumoural and non-tumoural exRNAs originate from a different organism background, enabling to distinguish these exRNA fractions using host-xenograft deconvolution algorithms on RNA sequencing data. Such algorithms are based on mapping of the sequencing reads to a combined host-xenograft reference genome or on parallel mapping to the host and xenograft genomes separately followed by filtering out misaligned reads. So far, several computational pipelines making use of these mapping strategies have been tested on tumour tissue of human xenograft mouse models and have been made available to the xenograft community. For example, Khandelwal *et al.* describe bamcmp for deconvolving host and graft reads in primary tumours and metastases from a melanoma mouse model, based on parallel mapping followed by a filtering approach based on the CIGAR string to discard reads misaligned to the host genome (24). Callari *et al.* use *in silico* combined human-mouse reference genome mapping (ICRG) of patient-derived breast cancer xenograft RNA sequencing data to accurately dissect the transcriptome of human tumour cells and mouse stroma (25). Kluin *et al.* report XenofilteR, which applies a parallel mapping strategy in combination with filtering based on the edit distance between a sequence read and a reference genome, and was evaluated on patient-derived xenograft (PDX) samples for which matched patient tumour samples were available (26). While these different methods have been evaluated on tumour-derived RNA, the performance of host-xenograft deconvolution algorithms on blood-derived exRNA currently remains unknown. This type of RNA is very different because of its extremely low concentration, highly fragmented nature and minority contribution of human tumour RNA relative to host RNA.

In this study, we investigated whether tumour-derived exRNA primarily resides in plasma fractions with higher platelet content, by means of total RNA sequencing of different blood fractions from 11 xenograft models, consisting of a human tumoural xenograft in a murine host; more precisely, 8 patient-derived xenograft (PDX) models and 3 cell line-derived xenograft (CDX) models. These models were analysed in two cohorts, a discovery cohort, which we present in more detail, and a validation cohort, of which the data are added as Supplementary Data. The discovery cohort consists of a breast cancer PDX model and a neuroblastoma SK-N-BE(2C) CDX model. The validation cohort includes seven PDX models (i.e. two endometrial cancers [EMC], three melanomas [MEL] and two penile cancers [PEN]) and three CDX models (i.e. one lung cancer [A549] and two neuroblastoma cancers (IMR-32 and SK-N-BE(2c)), the latter one being an expansion of the discovery cohort). First, two computational pipelines to reliably differentiate murine host and human tumour sequencing reads from plasma were optimized (exRNAxeno combined and parallel pipelines), demonstrating that read mapping to a combined reference genome is the best strategy for exRNA quantification in xenograft models. Subsequently, we analysed exRNA from platelets and three plasma fractions with different levels of platelets, obtained by applying successive centrifugation steps on murine blood. We show that murine platelets are not enriched for human tumour-derived RNA and that the circulating tumour signal is highly variable across xenograft models and individual mice. Highly abundant tumour transcripts in circulation correspond to high expression levels in tumour tissue, suggesting that circulating exRNA can be used as tumour biomarker in these models.

## MATERIALS AND METHODS

### Tumour tissue and liquid biopsy collection

The performance of the host-xenograft deconvolution algorithms was evaluated on liquid biopsies from non-tumour bearing control mice (TRACE PDX Platform, KU Leuven, UZ Leuven, Belgium) and human donors (ethical committee approval number EC/2017/1207, Ghent University Hospital, (27)). *In vivo* mice work was performed at the TRACE PDX platform (KU Leuven, approved by the local ethical committee for animal experimentation (P164/2019)). Female immunodeficient nude mice (NMRI-Foxn1^nu^ strain, Taconic Biosciences, Rensselaer, NY, USA, *n* = 8, of which five were used for the performance assessment of the pipelines and three for measuring platelet concentration) were subcutaneously injected with 30 μl of Roswell Park Memorial Institute (RPMI) 1640 medium (Thermo Fisher Scientific, Waltham, MA, USA), suspended in 70 μl Matrigel matrix (Corning, Bedford, MA, UK) at the age of 13 weeks. After 29–35 days, cardiac puncture was performed under anaesthesia to collect blood (volume range between 400 and 1000 μl), followed by cervical dislocation of the mice. The blood, collected in Microvette 500 K3EDTA tubes (Sarstedt, Newton, NC, USA), was processed immediately to complete blood-derived liquid biopsy preparation within 2 h after cardiac puncture. From each animal, three plasma fractions with different platelet content, i.e. single spun (SSP), double spun (DSP), and triple spun plasma (TSP), and platelets were prepared by means of three sequential centrifugation steps (details in Supplementary Figure S1), resulting in approximately 70 μl plasma per fraction. Throughout the manuscript, we make use of the term ‘liquid biopsy’ to refer to both plasma and platelets (3). Total RNA sequencing data from TSP samples of human donors (*n* = 2, two replicates for each donor) were obtained from Everaert *et al.* ((27); European Genome-Phenome Archive [EGA] sample ID EGAN00002518840-EGAN00002518843 in EGAS00001004428).

The discovery cohort consisted of two different xenograft mouse models, i.e. the BRC0004 patient-derived xenograft (PDX) mouse model (https://www.pdxfinder.org/data/pdx/TRACE/BRC0004) and an SK-N-BE(2C) cell line-derived xenograft (CDX) mouse model (TRACE PDX Platform; https://gbiomed.kuleuven.be/english/research/50488876/54502087/Trace/PDX-repository). To establish SK-N-BE(2C) CDX mice, female NMRI-Foxn1^nu^ mice (*n* = 5) were subcutaneously xenografted in the dorsal flank with 2 × 10^6^ SK-N-BE(2C) cells, in 30 μl RPMI 1640 medium (Thermo Fisher Scientific, Waltham, MA, USA), suspended in 70 μl Matrigel matrix (Corning, Bedford, MA, UK) at the age of 13 weeks. When CDX mice reached specific humane endpoints (i.e. a tumour volume of 2000 mm³, a weight loss of >20% or clinical signs of significant pain, distress or suffering), cardiac puncture was performed for liquid biopsy collection (as described above; Supplementary Figure S1; blood volume range between 250 and 1000 μl) followed by cervical dislocation. The BRC0004 PDX model was established from a tumour fragment freshly isolated from a triple-negative nodular breast invasive ductal adenocarcinoma patient by interscapular implantation in female NMRI-Foxn1^nu^ mice (*n* = 5). Tumours were propagated in at least three generations of mice and characterized by histology before being biobanked and used for this experiment. SNPs fingerprinting was used to confirm the genealogy before liquid biopsy collection. Liquid biopsies (SSP, DSP and TSP) of saline treated BRC0004 PDX mice were collected 3 weeks after the start of saline administration according to the protocol described in Supplementary Figure S2. From this xenograft model, also tumour tissue biopsies were collected and immediately stored in RNAlater (Thermo Fisher Scientific, Waltham, MA, USA) to be profiled using total RNA sequencing. For the CDX model, publicly available poly-A RNA sequencing data of the SK-N-BE(2C) cell line was used ((28); Gene Expression Omnibus [GEO] sample ID GSM2371256 in GSE89413). The validation cohort consisted of liquid biopsies from 20 additional PDX or CDX mice (neuroblastoma, melanoma, endometrial, penile and lung cancer xenograft models; details in Supplementary material and methods). All patients from whom tumour material was collected, provided informed consent prior to study participation, approved by the Ethical Committee UZ Leuven (S63799, S61605 and S66742).

The degree of haemolysis of all plasma samples was assessed by measuring levels of haemoglobin by spectrophotometric analysis (OD414) using a NanoDrop 1000 Spectrophotometer (Thermo Fisher Scientific, Waltham, MA, USA; Supplementary Tables S1 and 2). To confirm a reduction in platelet concentration upon successive centrifugation steps, platelet counts were measured in pooled SSP, DSP and TSP fractions of three non-tumour bearing female NMRI-Foxn1^nu^ mice, using an XN-1000 Haematology Analyzer (Sysmex, Kobe, Japan; impedance method, Supplemental Table S3).

### RNA isolation, spike-in RNA addition and DNase treatment

Extracellular RNA from 60 μl plasma or platelet pellets originating from 70 μl SSP, was isolated using the miRNeasy Serum/Plasma Kit (Qiagen, Hilden, Germany), according to the manufacturer’s manual. During RNA extraction, 2 μl of a 10 000-fold dilution of Sequin spike-in controls (Garvan Institute of Medical Research, Darlinghurst, NSW, Australia (29)) was added to the lysate. Upon RNA purification, 2 μl of a 25 000-fold dilution of External RNA Control Consortium (ERCC) RNA Spike-in Mix (Thermo Fisher Scientific, Waltham, MA, USA) was added to 12 μl RNA eluate, followed by gDNA removal (30). To this purpose, 1 μl HL-dsDNase (ArcticZymes Technologies, Tromsø, Norway) and 1.4 μl Heat & Run 10X Reaction Buffer (ArcticZymes Technologies, Tromsø, Norway) were added to the eluates, and RNA samples were incubated for 10 min at 37°C, followed by 5 min at 55°C.

RNA from tumour tissue was isolated using the miRNeasy Micro Kit (Qiagen, Hilden, Germany) in combination with the TissueLyser II system (Qiagen, Hilden, Germany). Upon RNA purification, 12 μl RNA eluate was used for gDNA removal as described above. DNase-treated RNA concentrations were measured by NanoDrop technology (Thermo Fisher Scientific, Waltham, MA, USA), and samples were diluted to 1.25 ng/μl using nuclease-free water (Saint Louis, MO, USA).

### RT-qPCR validation

For the validation of 2 Sequin spikes (R2_65 and R2_66) and 4 selected mouse platelet mRNAs (F5, Gng11, Nrgn, Ppbp), assays were carefully designed using the primer3plus tool (https://www.primer3plus.com with default settings, except amplicon size range of 60–100 nucleotides). The performance of the primers was thoroughly evaluated *in silico*. To determine the primer specificity, BiSearch (http://bisearch.enzim.hu with default settings, except for mismatch string, i.e. 1233333333333333) and the UCSC tool (https://genome.ucsc.edu/cgi-bin/hgPcr) were used. Subsequently, the OligoEvaluator tool (http://www.oligoevaluator.com/OligoCalcServlet) was used to check for secondary structure formation and GC content. Primers were ordered with Integrated DNA Technologies (IDT, Leuven, Belgium), purified by standard desalting. Primers were resuspended in TE buffer at 100 μM (10 mM Tris-HCl [pH 8.0], 0.1 mM EDTA) and stored at −20 °C. Primer efficiency and specificity were tested on a dilution series of mouse genomic DNA (Promega, G3091). To ensure that there is no cross-species amplification (i.e. murine primers amplifying human cDNA), we also checked for the absence of amplification on Universal Human Reference RNA (Agilent technologies, 750500), reverse transcribed to cDNA.

About 8 μl of DNase treated plasma RNA was reverse transcribed using the iScript Advanced cDNA Synthesis Kit for RT-qPCR (Bio-Rad, 1725038), according to the manufacturer’s manual. Subsequently, the cDNA was diluted 1:4, by adding 60 μl of nuclease free water to 20 μl cDNA and RT-qPCR was performed in a 5 μl reaction in duplicate in a 384-multiwell plate. Briefly, 2.5 μl of SsoAdvanced SYBR Green Supermix (Bio-Rad), 0.25 μl of each primer (5 μM) and 2 μl of diluted cDNA was added to each well, followed by a thermocycling protocol consisting of a preincubation step for 2 min at 95°C, followed by 44 amplification cycles (95°C for 5 s, 60°C for 30 s and 72°C for 1 s), ending with melt curve analysis during 1 s 0.11°C increment steps from 60 to 95°C on a LightCycler 480 system (Roche).

The *C*q values of the platelet genes were normalized by diminishing the mean *C*q of R2_65 and R2_66 spikes of each sample by the *C*q of each platelet gene of the respective sample.

### Total RNA library preparation and sequencing

Total RNA libraries were prepared starting from 8 μl DNase-treated RNA using the SMARTer Stranded Total RNA-Seq Kit v2 - Pico Input Mammalian (Takara Bio, CA, USA), according to the manufacturer’s manual with minor modifications (27). Briefly, prior to first strand cDNA synthesis, RNA from liquid biopsies and tumour tissue was fragmented for 2 min at 94°C. During final library amplification, 16 PCR cycles were performed on the liquid biopsy samples, while on tumour samples, only 13 cycles were performed. For the liquid biopsy samples, the final clean-up was repeated, since an excessive number of products <200 bp in size was observed on Fragment Analyzer data (data not shown, Agilent Technologies, Santa Clara, CA, USA). Fragment sizes were determined using Fragment Analyzer software for smear analysis in the 200 to 1000 bp range. Library quantification was performed using the KAPA Library quantification Kit (Kapa Biosystems, Wilmington, MA, USA) and libraries were pooled equimolarly. The final pool was quantified using Qubit, and 1.2 pM (for the PDX-derived samples) or 1.3 pM (for the CDX-derived samples) was loaded on a NextSeq 500 instrument (NextSeq 500 HighOutput Kit V2, 150 cycles), with 3% PhiX. Raw sequencing data is available in the European Genome-Phenome archive (EGAS00001005740 and EGAS00001006582).

### Preprocessing of RNA sequencing data

Sequencing reads of liquid biopsy and tumour samples were preprocessed by FastQC (v.0.11.8) for quality control and trimmed by Cutadapt (v.1.18) for low quality bases at the 3’ end of each read (Q30), for three nucleotides from the 5’ end of the second read (due to the template switching adapter) and for the HT-TruSeq adapter sequences. Reads <35 bp were filtered out. Next, duplicated reads were removed with Clumpify (BBMap v.38.26) within clumps based on 60 bp trimmed reads and using default parameters, except for 20 passes. To compare the different samples at similar sequencing depth, we downsampled (Seqtk v.1.3) to the lowest number of reads present within an experiment, i.e. 2223796 for the control and SK-N-BE(2C) CDX mice, and 4999120 for the BRC0004 PDX mice. To have sufficient sequencing depth remaining after downsampling, we removed one sample with very low sequencing depth, i.e. the TSP sample of control mouse CM9 (with only 1472031 reads; Supplementary Table S1). Subsequently, reads were once more analysed by FastQC for quality control. QC analyses resulted in the exclusion of all samples from control mouse CM11, since these samples displayed short read sequences (<70 bp) and high levels of trimming (34.6–52.8%; Supplementary Table S1) compared to the other control mouse samples.

### exRNAxeno computational framework for combined and parallel mapping of RNA sequencing data

Reads were mapped using STAR (v.2.6.0) (specific parameter settings: –outSAMprimaryFlag AllBestScore –outSAMattributes NH HI AS nM NM) to either a combined reference genome of human and mouse (combined mapping) or in parallel to both the human and mouse genome (parallel mapping). The genome index for mapping was built using the Ensembl GRCh38.94 (human) and GRCm38.94 (mouse) DNA primary assembly sequences, containing all chromosomes, the mitochondrial genome and scaffolds, supplemented with ERCC and Sequin spike sequences and the full ribosomal DNA complete repeating unit (U13369.1, BK000964.3). GTF files were downloaded from Ensembl and adapted in a similar way. In the combined reference genome of mouse and human, mouse chromosomes were labelled with a prefix ‘m’. For both the combined and parallel mapping, uniquely mapped reads were selected based on the NH:i:1 tag (SAMtools v.1.8, Pysam). In the parallel mapping, read pairs mapping to both human and mouse were assigned to the organism where mapping resulted in the smallest edit distance (i.e. sum of NM tag and CIGAR string soft-clipping from both read pairs in the SAM file; Pysam, Picard v.2.21.1). In both combined and parallel mapping, BAM files were further masked by intersectBed for regions where control murine (*n* = 15) and human (*n* = 4) liquid biopsies empirically showed misalignment to the other reference genome (SAMtools v.1.8, BEDtools v.2.27.1, BEDOPS v.2.4.32). Further quality control on the filtered BAM files was done using MultiQC (v.1.7), SAMtools (v.1.8), RseQC (v.2.6.4) and BEDTools (v.2.27.1). Finally, read counts of name-sorted BAM files were generated by HTSeq-count (v.0.11.0) (specific parameter settings: -s reverse –secondary-alignments = ignore –supplementary-alignments = ignore) using appropriate GTF files. Further processing was done with R (v.4.0.3) making use of tidyverse (v.1.3.1). The exRNAxeno combined and exRNAxeno parallel pipeline are available through GitHub (https://github.com/CBIGR/exRNAxeno).

### Determination of human and mouse RNA concentration in liquid biopsies using RNA spike-in sequencing data

The mass of endogenous RNA present in 1 ml of plasma was determined based on the known amount of Sequin spike-in controls added during RNA isolation (as described in (30)). First, the mass of all Sequin spike-in controls was calculated, based on the length (in nucleotides) and molar concentration (in attomol/μl) of each Sequin spike-in control (Supplementary Table S4). By multiplying the concentration (in attomol/μl) with the volume of spikes added (2 μl) and the dilution factor (1/10 000), the mass amount (attomol) of each Sequin spike-in control added to the sample was calculated. Second, to convert the amount (attomol) to weight (g), the molecular weight of each Sequin spike-in control was determined, by multiplying its length (nt) with the average molecular weight of a single nucleotide (321.47 g/mol). Next, individual Sequin spike-in control weights were summarized to obtain a total weight of 1.32E-12 g. For the SSP/DSP/TSP samples (with an input volume of 60 μl plasma), this resulted in 21.94 pg Sequin spike-in control added per mL of plasma, while for the platelets (originating from 70 μl SSP), this equaled to 18.81 pg Sequin spike-in controls per platelet lysate. Finally, by multiplying the endogenous RNA over Sequin spike in-control read count ratio with the amount of Sequin spike-in control added per fraction (pg/mL), the endogenous RNA concentration was obtained (pg/mL).

### Statistical analysis

To compare the combined and parallel pipelines, we applied a non-parametric two-tailed Wilcoxon rank-sum test using normal approximation on the fraction of misaligned reads. For differential expression analysis between engrafted and non-engrafted mice, RNA sequencing reads were first normalized using the median sum of the Sequin spike-in read counts. Unexpressed genes were excluded from the analyses. Next, ANOVA comparisons were performed using scipy statistics (v.1.1.0) and visualization was performed using seaborn clustermap (v.0.9.0), both in Python. Data were visualized in heatmaps using ward-clustering. *P*-values were calculated using a Fisher’s exact test in R (multiple testing correction not performed). To compare the normalized exRNA concentrations and the percentage of tumoral exRNA in the different plasma fractions, the non-parametric Friedman chi-square test for repeated measurements was carried out, followed by the post-hoc Nemenyi all-pairs test with Benjamini–Hochberg multiple hypothesis correction. A two-sample Kolmogorov–Smirnov test investigated if there was a difference in the cumulative abundance of genes detected in the plasma or not, based on their abundance in the tumour tissue.

## RESULTS

### Read mapping on a combined reference genome is the preferred strategy for the analysis of extracellular RNA from xenograft-derived liquid biopsies

**Figure 1.**
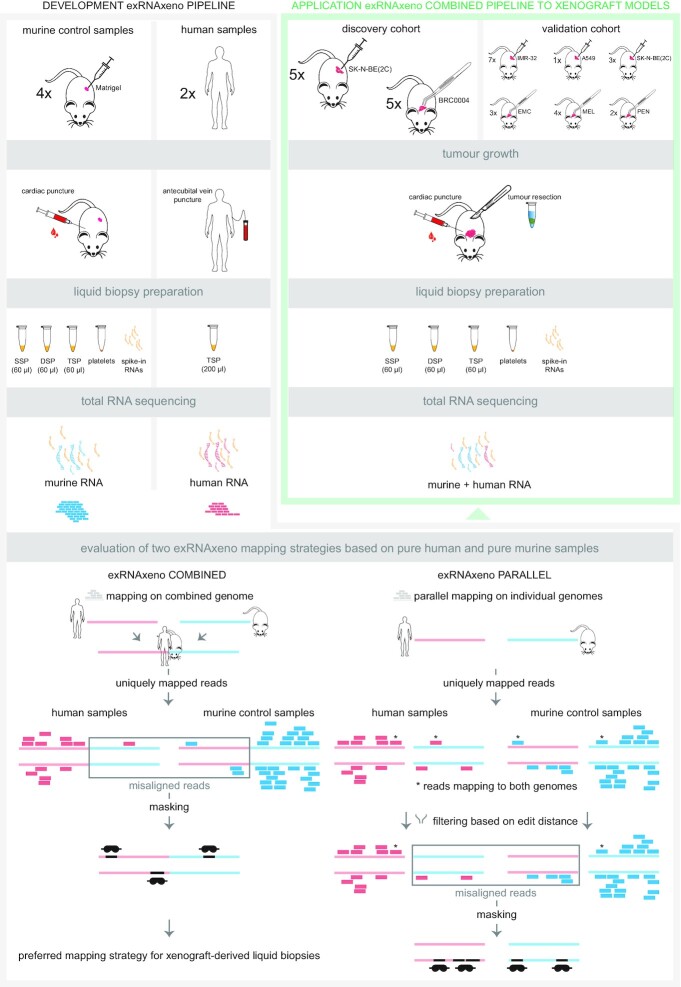
Development and application of a deconvolution pipeline applicable to liquid biopsies from human tumour xenografts. First, both the exRNAxeno combined and parallel mapping analysis pipeline were applied to total RNA sequencing data from pure (non-xenograft) exRNA samples from liquid biopsies (i.e. blood platelets, single spun [SSP], double spun [DSP] and triple spun plasma [TSP]) of mice without tumour xenograft, and from TSP of human donors, in order to characterize and mask genomic regions containing misaligned reads. Subsequently, the optimized exRNAxeno combined mapping pipeline was applied to total RNA sequencing data of exRNA from three different plasma fractions (SSP, DSP and TSP), and platelets from two different xenograft mouse cohorts, a discovery and validation cohort, in order to characterize the human tumoural RNA signal in the murine blood; EMC, endometrial cancer; MEL, melanoma; PEN, penile cancer.

**Figure 2.**
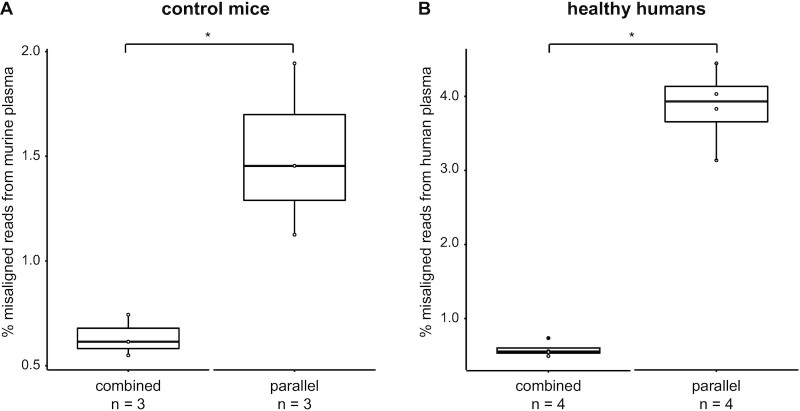
The exRNAxeno combined mapping pipeline results in significantly lower fractions of misaligned reads from both murine and human triple spun plasma. (**A**) Percentage of misaligned reads from murine plasma for both the exRNAxeno combined and parallel mapping pipeline (non-parametric two-tailed Wilcoxon rank-sum test using normal approximation, *P* = 0.049 (*)). (**B**) Percentage of human reads misaligning to the mouse genome for both the exRNAxeno combined and parallel mapping pipeline (non-parametric two-tailed Wilcoxon rank-sum test using normal approximation, *P* = 0.021 (*)). * refers to a significance of *P* < 0.05.

Both combined and parallel mapping approaches are being used for the computational deconvolution of a human tumour xenograft transcriptome in mice. Due to the high homology between human and mouse, these computational approaches result in a limited number of misaligned reads, i.e. false positive RNA signals in either species. Here, we developed and compared two computational pipelines to accurately distinguish between tumour-derived (human origin) and non-tumoural (mouse origin) exRNA in liquid biopsies from human tumour xenograft mouse models: one using a combined mapping approach (exRNAxeno combined) where reads are mapped to a combined reference genome of human and mouse, and one using a parallel mapping strategy (exRNAxeno parallel) where reads are mapped to the human and murine genome separately and subsequently assigned as being either human or murine based on comparison of the edit distances (Figure 1, see Materials and Methods). The performance of both computational strategies was evaluated by charting the misaligned reads in total RNA sequencing data sourced from pure murine exRNA from blood platelets, single spun (SSP), double spun (DSP) and triple spun plasma (TSP) of control mice, i.e. mice without tumour xenograft of human origin, and of pure human exRNA from TSP from healthy human donors. Both the combined and parallel mapping pipeline detected reads in murine plasma misaligned to the human genome and reads in human plasma misaligned to the murine genome. More precisely, in TSP, 0.5–0.7% and 1.1–1.9% of the murine plasma reads is misaligned to the human genome in the combined mapping and parallel mapping strategy, respectively (Figure 2, non-parametric two-tailed Wilcoxon rank-sum test using normal approximation, *P* = 0.049). Reversely, also mapping of the human plasma reads to the murine genome leads to misaligned reads (0.5–0.7% in the combined mapping and 3.1–4.5% in the parallel mapping, non-parametric two-tailed Wilcoxon rank-sum test using normal approximation, *P* = 0.021). At the gene level, this corresponds to 93 and 314 unique human Ensembl gene IDs that are robustly detected (i.e. ≥ 5 read counts) in the murine plasma samples by the combined and parallel mapping pipeline, respectively. In the human plasma samples, we identified 24 and 60 unique murine Ensembl gene IDs, respectively. Most of these misaligned genes correspond to repetitive or highly homologous sequences (https://github.com/CBIGR/exRNAxeno). To further optimize the exRNAxeno framework, we used the misaligned reads in both the human and murine control samples to mask the genome in the analysis of tumour bearing mice samples, as these regions seem to be too homologous to differentiate even with stringent mapping conditions. This resulted in 60 259 720 bp (0.6% of genome sequence) masked from the human genome part and 12 640 841 bp (0.2%) masked from the murine genome part in the combined mapping strategy. In the parallel mapping strategy, 229 383 813 bp (2.3%) of the human genome and 41 627 990 bp (0.7%) of the murine genome are masked. Reprocessing of the human and control murine samples with the masking filter active completely removed the misaligned reads and cross-reference gene IDs (Supplementary Figure S3). In conclusion, as the combined mapping strategy resulted in significantly lower fractions of misaligned reads (Figure 2) and fewer false positive Ensembl gene IDs, and required a smaller masked region, we put forward exRNAxeno combined as the preferred computational pipeline for the analysis of exRNA from xenograft-derived liquid biopsy samples.

### The tumoural RNA concentration is not determined by the platelet level in plasma

**Figure 3.**
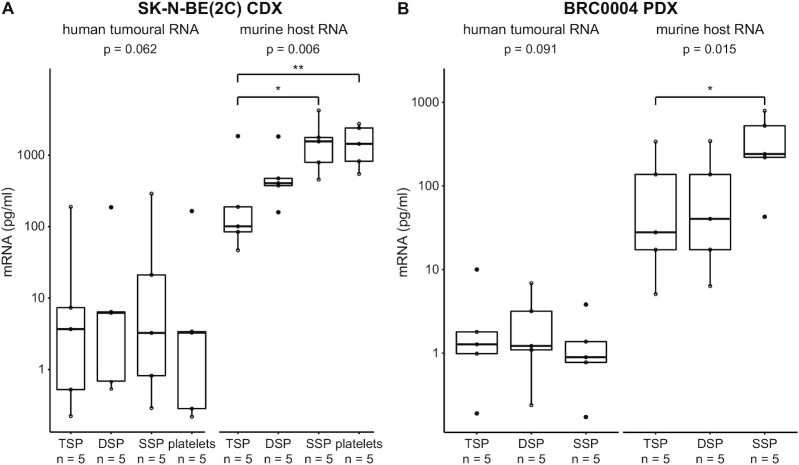
The tumoural exRNA concentration is relatively constant across the different liquid biopsies, in contrast to the host exRNA concentration. (**A**) Tumour and host exRNA concentrations in each plasma fraction and in platelet pellets from the cell line-derived xenograft (CDX) mice (Friedman chi-squared test = 12.6, df = 3, *P* = 0.006; post-hoc test Nemenyi, *P* = 0.017 (*) and *P* = 0.008 (**)). (**B**) Tumour and host exRNA concentrations in each plasma fraction from the patient-derived xenograft (PDX) mice (Friedman chi-squared test = 8.4, df = 2, *P* = 0.015; post-hoc test Nemenyi, *P* = 0.012 (*)). DSP, double spun plasma; SSP, single spun plasma; TSP, triple spun plasma. * refers to a significance of*P* < 0.05; ** refers to a significance of *P* < 0.01.

**Figure 4.**
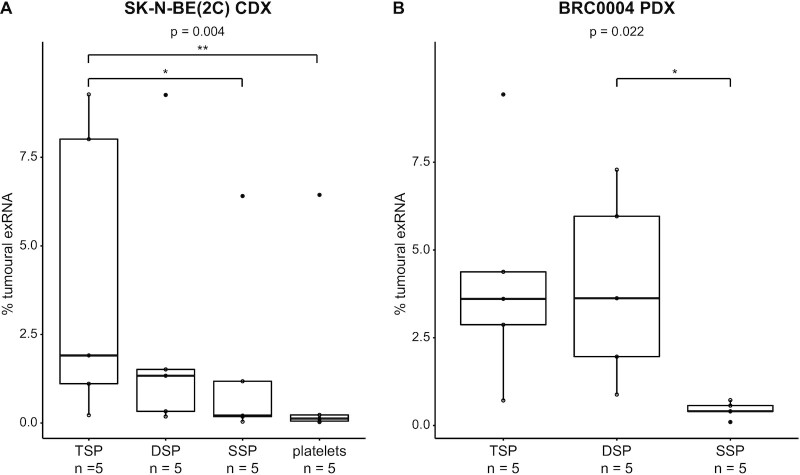
The tumoural exRNA percentage is inversely proportional to the platelet plasma level. Shown are the percentages of tumoural exRNA present in the different liquid biopsies from the CDX (**A**; Friedman chi-squared test = 13.56, df = 3, *P* = 0.004; post-hoc test Nemenyi, *P* = 0.036 (*) and *P* = 0.017 (**)) and PDX (**B**; Friedman chi-squared test = 7.6, df = 2, *P* = 0.022; post-hoc test Nemenyi, *P* = 0.031 (*)) mice. DSP, double spun plasma; SSP, single spun plasma; TSP, triple spun plasma. * refers to a significance of*P* < 0.05; ** refers to a significance of *P* < 0.01.

Next, the optimal computational pipeline, i.e. exRNAxeno combined, was applied to total RNA sequencing data from liquid biopsy exRNA from two human xenograft mouse models, i.e. SSP, DSP, TSP and platelets from a breast cancer patient-derived (BRC0004, PDX) and neuroblastoma cell line-derived (SK-N-BE(2C), CDX) xenograft mouse model (Figure 1 and Supplementary Table S1). Before assessing differences between the different blood fractions, differential abundance analyses were performed between tumour-bearing and non-tumour-bearing mice, hinting towards differential exRNA abundance profiles between engrafted and non-engrafted mice (Supplemental Figure S4). Next, we investigated the human tumoural and murine host signal in the different blood fractions of the xenografted mice, by quantifying exRNA concentrations using synthetic Sequin spike-in RNAs, added during exRNA purification (see Materials and Methods). RT-qPCR validation of Sequin spikes (R2_65 and R2_66) in the eluates showed that exRNA purification is reproducible among samples and that Sequin spike-in RNA can be used for quantification of exRNA concentrations (Supplementary Figure S5, Pearson’s correlation coefficient of 0.6531 and *P* < 0.05 between Cq values of R2_65 and R2_66). Successive centrifugation steps resulted in lower plasma host RNA concentration in both the CDX (Friedman chi-squared test ( = 12.6), df = 3, *P* = 0.006) and PDX experiment (Friedman chi-squared test ( = 8.4), df = 2, *P* = 0.015) (Figure 3). Gene set enrichment analyses (GSEA) on differentially abundant host genes between SSP, DSP and TSP demonstrated that this host RNA signal is dominated by platelet specific transcripts (Supplementary Table S5), in line with the fact that successive centrifugation resulted in a gradual decrease in plasma platelet concentration. This gradual decrease in platelet concentration was confirmed by platelet counts on pooled SSP, DSP and TSP from three control mice (Supplementary Table S3) and by RT-qPCR validation of platelet mRNA markers (F5, Gng11, Nrgn and Ppbp; Supplementary Figure S6). We were also able to detect tumoural exRNA in all plasma fractions and in platelets from the xenografted mice, but amounts are highly variable across individual animals (Figures 3, 4 and Supplementary Figure S7). More precisely, 0.03–9.27% and 0.10–9.43% of the exRNA is tumour-derived in the CDX and PDX mice, respectively (Figure 4 and Supplementary Figure S7). Remarkably, while we observed a gradual decrease in host RNA concentration with decreasing platelet content, tumoural RNA concentration levels are comparable across the different liquid biopsy types in both experiments (Figure 3). As a result, TSP samples contain the highest fraction of tumour-derived exRNAs, due to the lower background of host exRNAs (Figure 4 and Supplementary Figure S7).These findings have been confirmed in a validation cohort comprising of liquid biopsies from 20 additional CDX and PDX mice (neuroblastoma, melanoma, endometrial, penile and lung cancer xenograft models) using the same workflow technology as used in the discovery cohort (Supplementary Materials and Methods, Supplementary Figure S8).

### The circulating tumour transcriptome is highly variable across xenografted mice

**Figure 5.**
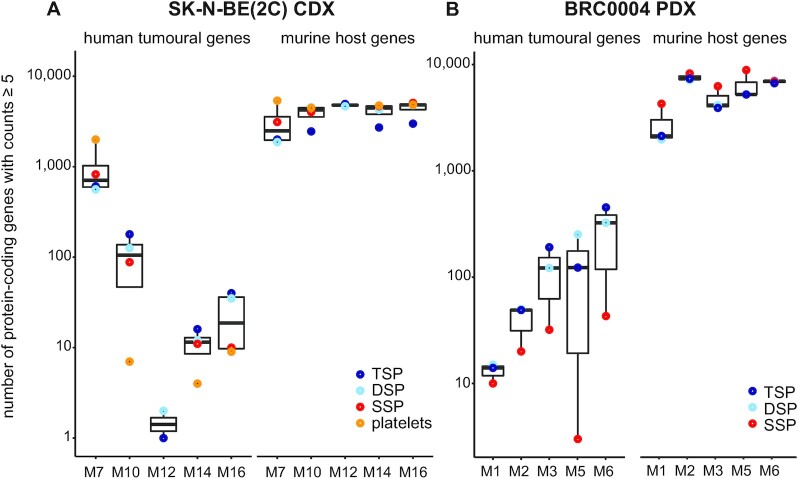
The number of host and tumour protein-coding genes varies across xenografted mice. Shown are the numbers of robustly detected protein-coding genes (i.e. ≥5 counts) in the individual CDX (**A**) or PDX (**B**) mice. The different colours represent the different liquid biopsies; DSP, double spun plasma; SSP, single spun plasma; TSP, triple spun plasma.

**Figure 6.**
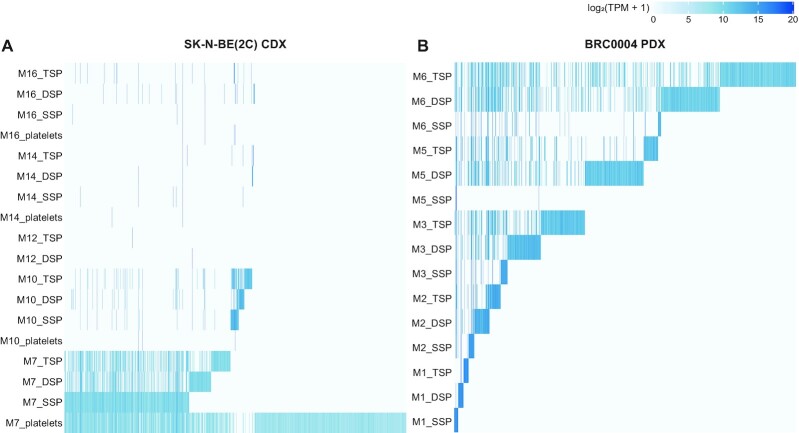
The circulating tumour gene abundance profile is highly variable across individual mice. Log_2_(TPM + 1) values of all circulating protein-coding genes robustly detected (i.e. ≥5 counts) in at least one liquid biopsy sample of the CDX (**A**) or PDX (**B**) mice; DSP, double spun plasma; SSP, single spun plasma; TSP, triple spun plasma.

**Figure 7.**
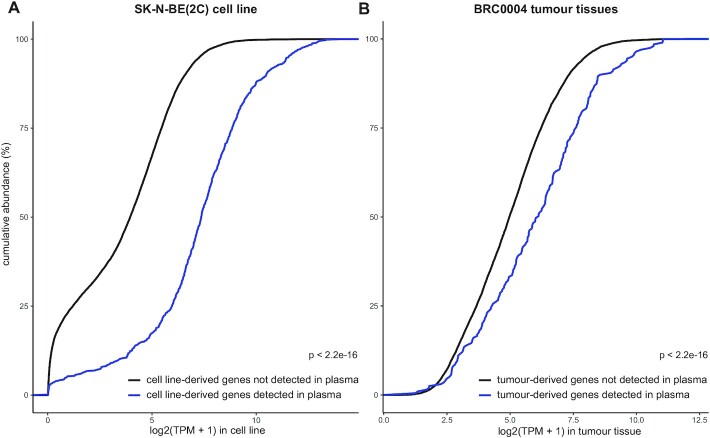
The tumour-derived genes that are circulating in plasma are higher expressed in the originating CDX or PDX cancer cells than the tumour-derived genes that are not circulating in plasma. (**A**) Cumulative abundance of genes only detected in the cell line (black) and genes detected in both the cell line and plasma (blue) of the CDX mice (two-sample Kolmogorov–Smirnov test, *P* < 2.2e-16). (**B**) Cumulative abundance of genes only detected in the tumour tissue (black) and genes detected in the tumour tissue and plasma (blue) of the PDX mice (two-sample Kolmogorov–Smirnov test, *P* < 2.2e-16). Log_2_(TPM+1) in tumour tissue represents the mean of the log_2_(TPM+1) tumour tissue values of mice M3, M5 and M6. A tumour gene is considered to be circulating in plasma if it has ≥ 5 counts in at least one of the triple spun plasma (TSP) samples.

Although multiple RNA biotypes are detected in the liquid biopsies, the majority of detected tumour-derived Ensembl gene IDs is protein-coding, i.e. 94.4% in the CDX and 73.2% in the PDX mice samples (Supplementary Figure S9). When focusing on the protein-coding gene fraction, the number of robustly detected host-derived genes in circulation is on average 921 times (ranging from 3 to 5381 times, CDX mice samples) or 326 times (ranging from 15 to 2960 times, PDX mice samples) higher than the number of tumour-derived genes (Figure 5). In general, TSP has the highest number of human tumoural and lowest number of murine host genes, while SSP and platelets show the opposite pattern (Figures 5 and 6). This could also be confirmed in the xenograft models of the validation cohort (Supplemental Figure S10A–F). Although differences between liquid biopsy types can be observed, differences across individual mice are more pronounced (Figures 5 and 6). More specifically we detected 2126 (M7), 244 (M10), 75 (M16), 32 (M14) or 3 (M12) human tumoural protein-coding genes in total, across all liquid biopsy types in the CDX mice, and 612 (M6), 315 (M5), 270 (M3), 91 (M2) or 37 (M1) human tumoural protein-coding genes in the PDX mice. Although sample-specific gene sets largely determine the circulating tumour transcriptomes of the xenograft mice, common gene sets both across different liquid biopsy types and—to a lesser extent—across different mice can be observed (Figure 6, Supplementary Figures S10A–F and 11). The large differences in the number of tumour-derived genes across individual mice cannot be correlated to PDX and CDX tumour volumes, since tumour volumes did not differ across individual PDX (tumour volume range: 1104.1–1286.7 mm³) and CDX (1962.8–2115.1 mm³) mice. Next, we investigated the expression levels of tumoural protein-coding genes in total RNA sequencing data of the PDX tumour tissue biopsies and publicly available polyA+ RNA sequencing data of SK-N-BE(2C) cells and linked this to the detectability of these genes in circulation. Given the high variability in the number of tumour-derived genes among the xenografted mice (Figure 6), these analyses were confined to the TSP samples from a few mice with the highest number of protein-coding genes detected, i.e. CDX mice M7 and M10, and PDX mice M3, M5 and M6. As depicted in Figure 7, tumour genes in circulation are significantly higher expressed in the tumour tissue (PDX) or cell line (CDX) than tumour genes not detected in circulation (two-sample Kolmogorov–Smirnov test, *P* < 2.2e-16).

## DISCUSSION

To assess whether blood platelets from cancer patients are preferentially loaded with tumoural RNA, we charted the tumour-derived transcriptome in different types of liquid biopsies from two human xenograft mouse models. Using such models, the circulating human, tumoural transcriptome can be differentiated from the murine, non-tumoural extracellular RNAs using host-xenograft deconvolution algorithms for RNA sequencing read analysis. Although various host-xenograft deconvolution pipelines have been described, either based on a parallel mapping and filtering strategy or a combined mapping strategy (24–26,31–33), they have not been extensively compared and underlying bioinformatics pipelines are generally not publicly available. More importantly, these pipelines have not been applied to challenging blood-derived exRNA sequencing data, which is vastly different because of low concentration and fragmented nature. In the first part of this study, we developed and evaluated the performance of two deconvolution pipelines, using a combined (exRNAxeno combined) or parallel (exRNAxeno parallel) mapping strategy, on total exRNA sequencing data of liquid biopsies from non-tumour bearing control mice and healthy human donors. It should be noted that transcriptome profiling was performed using a total library preparation kit that can handle very low input amounts of fragmented RNA from different mammalian organisms and that the performance of the applied RNA sequencing technology has been previously validated (27). Here, exRNA from only 60 μl of plasma was used for sequencing library preparation. Both the exRNAxeno combined and exRNAxeno parallel pipeline demonstrated good performance and were further fine-tuned by masking genomic regions in which misaligned reads were identified in the control samples. By providing the computational pipelines through GitHub (https://github.com/CBIGR/exRNAxeno), we supply the research community with important tools to further explore circulating (tumour) transcriptomes in xenograft models. In addition, all sequencing data have been submitted to the European Genome-phenome Archive (EGA accession ID: EGAS00001005740 and EGAS00001006582). Given that the exRNAxeno combined mapping pipeline resulted in lower fractions of misaligned reads, fewer false positive Ensembl gene IDs and a smaller masked region, this method is preferred for the analysis of liquid biopsies from human xenograft mouse models.

Subsequently, our exRNAxeno combined pipeline was applied to exRNA samples from two xenograft mouse models, i.e. exRNA from platelets and three plasma fractions with decreasing platelet numbers (SSP, DSP and TSP) from SK-N-BE(2C) CDX and BRC0004 PDX mice, enabling to study the impact of plasma platelets levels on the circulating tumoural exRNA concentration and gene content. Finally, results were validated in 10 xenograft models, including neuroblastoma, melanoma, endometrial, penile and lung cancer xenograft models. Although the numbers of animals of the different xenograft models are limited, findings were consistent across the different xenograft models. We observed a gradual decrease in host RNA concentration with decreasing platelet content, while the tumoural RNA concentration remained relatively constant. This points out that murine platelets do contain a significant amount of murine exRNA, but that the tumoural RNA does not primarily reside in platelets. Of note, in contrast to the SK-N-BE(2C) CDX model, host RNA concentration levels in the BRC0004 PDX DSP and TSP samples do not differ (Figures 3 and 4), which may be explained by the different plasma preparation protocol that was used for these two xenograft models. In the PDX model, a longer centrifugation time (15 min versus 10 min in the CDX model) and higher *g*-force (2500 *g* versus 800 *g* in the CDX model) was used to prepare DSP, resulting in removal of most of the platelets in the PDX DSP sample, and as such comparable platelet levels between the PDX DSP and TSP samples (as confirmed by the GSEA and RT-qPCR results, Supplementary Table S5 and Supplementary Figure S6). This clearly demonstrates that the plasma preparation protocol in exRNA-based tumour xenograft studies has substantial impact on the amount of background host signal that is detected, and thus also the tumoural exRNA proportion (Figure 4 and Supplementary Figure S8). Since platelets or platelet-rich plasma fractions are not enriched for tumour genes (Figure 5) and platelet-deprived plasma fractions display a greater tumour gene diversity (Figure 6 and Supplementary Figure S10A–F), we put forward TSP samples as the preferred biomaterial for tumour extracellular transcriptome profiling using massively parallel sequencing. Moreover, the greater tumour gene diversity from platelet-deprived fractions allows for enhanced detection of tumour aberrations. However, although TSP holds a greater tumour gene diversity, this does not preclude that the abundance of specific tumour genes might be higher in other plasma fractions. Future research should confirm these findings in biofluids from cancer patients (e.g. by focusing on characteristic genetic defects of the tumour, such as somatic mutations), but we expect that platelet depletion is also preferred when working with human plasma, in order to minimize the background signal originating from non-tumoural platelet RNA. We did not study possible enrichment of tumoural RNA in other plasma fractions such as extracellular vesicles (EVs) as this was practically impossible given the low starting volume (i.e. 60 μl) of plasma. Ultrapure purification of EVs cannot be performed on such small volumes and anticipating that EV RNA concentrations will be much lower than total plasma RNA concentrations, we would probably have reached the detection limit of total RNA sequencing (27). Unfortunately, the low number of mice per model and an insufficient sequencing depth did not allow us to analyse differential splicing patterns of platelets between tumour-bearing mice and non-tumour bearing mice, as reported for so-called tumour-educated platelets (15,16,20).

Of note, the number of detected genes and RNA concentration as reported here depend on many factors and should thus primarily be used for comparisons in this study. Apart from the plasma preparation protocol, also other factors influence exRNA profiles, such as the type of blood collection tube and the volume of plasma used for exRNA purification. In mice, the maximum volume of blood that can be collected by cardiac puncture is approximately 1 ml. By focusing on a single plasma fraction in these models, or by making use of xenograft models in larger animals (with higher blood volumes), larger plasma volumes can be obtained, which could result in higher yields of purified exRNA. Other factors that determine exRNA analysis are the RNA purification and sequencing library preparation method, as well as the exRNA eluate fraction used as input for library preparation (34). On top of that, also sequencing depth and specific analysis settings (e.g. the read count cut-off for robust detection) impact results. Clearly, the impact of these pre-analytical and analytical variables on tumour exRNA profiles should be further investigated, with the aim to develop guidelines for tumour exRNA analysis, enabling to compare results from different studies and to implement the use of exRNA-based biomarkers in oncology. Compared to the steps taken to standardize circulating tumour DNA (ctDNA) analysis, the efforts made to setup standard operating procedures for tumour exRNA analysis are still rather limited (35). Nevertheless, exRNAs are promising precision oncology biomarkers that expand the horizons of tumour-derived nucleic acid analysis. For example, it has been previously shown that combined DNA and RNA analyses from plasma result in higher detection rates of EGFR mutant lung cancer (21). This example also underscores the need to further investigate the parallel analysis of tumour exRNA and other blood analytes (such as ctDNA).

Apart from differences across the plasma fractions, individual mice present with even larger differences. Genuine biological variation, rather than technical variation, likely underlies these differences as our data is shown to be of good quality. The quality of the sequencing reads and the preprocessing steps was assured by FastQC and MultiQC analyses; the relatively constant Sequin/ERCC spike-in RNA ratio and the RT-qPCR spike-in RNA measurements (Supplementary Figure S5) all demonstrate that experiments are well executed (Supplementary Tables S1 and 2) (30). In search of tumour features that may explain the observed differences in the number of circulating tumour genes, we assessed the PDX and CDX tumour volumes. Since tumour volumes in PDX and CDX mice do not differ across individual mice, the number of circulating tumour genes is not associated with tumour volume. Also, our results do not point towards preferential shedding of certain tumoural exRNA in circulation, as highly abundant tumour genes in circulation are generally highly expressed in the originating tumour tissue or cell line.

In conclusion, we provide a novel computational framework, exRNAxeno, for the analysis of exRNA in plasma from tumour xenograft models, which enables to distinguish tumoural exRNAs from host background exRNA. Using this workflow, we characterized the entire extracellular tumour transcriptome in different plasma fractions of various human patient tumour-derived and cellular-derived murine xenograft models. As such, we demonstrated that the tumoural RNA concentration is not determined by the blood platelet level in plasma, and that the circulating tumour transcriptome is highly variable across individual xenograft mice. In general, highly abundant tumour-derived transcripts in plasma also display high expression levels in the tumours. These findings open new avenues to further investigate the functional role of circulating exRNAs in cancer models and patients.

## DATA AVAILABILITY

The datasets supporting the conclusions of this article are available on the European Genome-Phenome Archive (EGA). Total RNA sequencing data from TSP samples of human donors (*n* = 2, two replicates for each donor) were obtained from Everaert *et al.* (27); European Genome-Phenome Archive (EGA) sample ID EGAN00002518840-EGAN00002518843 in EGAS00001004428). Total RNA sequencing data from the control mice, the BRC0004 PDX mouse model, the SK-N-BE(2C) CDX mouse model and PDX/CDX validation cohort have been deposited in EGA (EGAS00001005740 and EGAS00001006582). The exRNAxeno combined and parallel pipeline for processing of RNA sequencing data are available in the GitHub repository (https://github.com/CBIGR/exRNAxeno).

## Supplementary Material

zcac037_Supplemental_Files

## References

[1] Suraj S. , Dhar C., Srivastava S. Circulating nucleic acids: an analysis of their occurrence in malignancies (review). Biomed. Rep. 2017; 6:8–14. 28123700 10.3892/br.2016.812 PMC5244781

[2] Esposito A. , Criscitiello C., Locatelli M., Milano M., Curigliano G. Liquid biopsies for solid tumors: Understanding tumor heterogeneity and real time monitoring of early resistance to targeted therapies. Pharmacol. Ther. 2016; 157:120–124. 26615782 10.1016/j.pharmthera.2015.11.007

[3] Heitzer E. , Roberts C.E.S., Speicher M.R. Current and future perspectives of liquid biopsies in genomics-driven oncology. Nat. Rev. Genet. 2019; 20:71–88. 30410101 10.1038/s41576-018-0071-5

[4] Bronkhorst A.J. , Ungerer V., Holdenrieder S. the emerging role of cell-free DNA as a molecular marker for cancer management. Biomol. Detect. Quantif. 2019; 17:100087. 30923679 10.1016/j.bdq.2019.100087 PMC6425120

[5] Hulstaert E. , Morlion A., Avila Cobos F., Verniers K., Nuytens J., vanden Eynde E., Yigit N., Anckaert J., Geerts A., Hindryckx P. et al. Charting Extracellular Transcriptomes in the Human Biofluid RNA Atlas. Cell Rep. 2020; 33:108552. 33378673 10.1016/j.celrep.2020.108552

[6] Zhou Z. , Wu Q., Yan Z., Zheng H., Chen C.J., Liu Y., Qi Z., Calandrelli R., Chen Z., Chien S. et al. Extracellular RNA in a single droplet of human serum reflects physiologic and disease states. Proc. Natl. Acad. Sci. U.S.A. 2019; 116:19200–19208. 31481608 10.1073/pnas.1908252116 PMC6754586

[7] Zeka F. , Decock A., van Goethem A., Vanderheyden K., Demuynck F., Lammens T., Helsmoortel H.H., Vermeulen J., Noguera R., Berbegall A.P. et al. Circulating microRNA biomarkers for metastatic disease in neuroblastoma patients. JCI Insight. 2018; 3:e97021. 30518699 10.1172/jci.insight.97021 PMC6328024

[8] Mithraprabhu S. , Morley R., Khong T., Kalff A., Bergin K., Hocking J., Savvidou I., Bowen K.M., Ramachandran M., Choi K. et al. Monitoring tumour burden and therapeutic response through analysis of circulating tumour DNA and extracellular RNA in multiple myeloma patients. Leukemia. 2019; 33:2022–2033. 30992504 10.1038/s41375-019-0469-x

[9] D’Ambrosi S. , Nilsson R.J., Wurdinger T. Platelets and tumor-associated RNA transfer. Blood. 2021; 137:3181–3191. 33940602 10.1182/blood.2019003978 PMC8351881

[10] McAllister S.S. , Weinberg R.A. the tumour-induced systemic environment as a critical regulator of cancer progression and metastasis. Nat. Cell Biol. 2014; 16:717–727. 25082194 10.1038/ncb3015 PMC6220424

[11] Calverley D.C. , Phang T.L., Choudhury Q.G., Gao B., Oton A.B., Weyant M.J., Geraci M.W. Significant downregulation of platelet gene expression in metastatic lung cancer. Clin. Transl. Sci. 2010; 3:227–232. 21500395 10.1111/j.1752-8062.2010.00226.x PMC3427741

[12] Xue L. , Xie L., Song X., Song X. Identification of potential tumor-educated platelets RNA biomarkers in non-small-cell lung cancer by integrated bioinformatical analysis. J. Clin. Lab Anal. 2018; 32:e22450. 29665143 10.1002/jcla.22450 PMC6817076

[13] Nilsson R.J.A. , Balaj L., Hulleman E., van Rijn S., Pegtel D.M., Walraven M., Widmark A., Gerritsen W.R., Verheul H.M., Vandertop W.P. et al. Blood platelets contain tumor-derived RNA biomarkers. Blood. 2011; 118:3680–3683. 21832279 10.1182/blood-2011-03-344408 PMC7224637

[14] Liu L. , Lin F., Ma X., Chen Z., Yu J. Tumor-educated platelet as liquid biopsy in lung cancer patients. Crit. Rev. Oncol. Hematol. 2020; 146:102863. 31935617 10.1016/j.critrevonc.2020.102863

[15] Sol N. , in ‘t Veld S.G.J.G., Vancura A., Tjerkstra M., Leurs C., Rustenburg F., Schellen P., Verschueren H., Post E., Zwaan K. et al. Tumor-educated platelet RNA for the detection and (pseudo)progression monitoring of glioblastoma. Cell Rep. Med. 2020; 1:100101. 33103128 10.1016/j.xcrm.2020.100101 PMC7576690

[16] Heinhuis K. , in ’t Veld S., Dwarshuis G., van den Broek D., Sol N., Best M., Koenen A., Steeghs N., Coevorden F., Haas R. et al. RNA-sequencing of tumor-educated platelets, a novel biomarker for blood based sarcoma diagnostics. Eur. J. Surg. Oncol. 2020; 46:e7. 32471035 10.3390/cancers12061372 PMC7352477

[17] Roweth H. , Battinelli E. Lessons to learn from tumor-educated platelets. Blood. 2021; 137:3174–3180. 33940594 10.1182/blood.2019003976 PMC8351883

[18] Jia J. , Yang S., Huang J., Zheng H., He Y., Wang L. Distinct extracellular RNA Profiles in different plasma components. Front. Genet. 2021; 12:564780. 34234804 10.3389/fgene.2021.564780 PMC8256274

[19] Brinkman K. , Meyer L., Bickel A., Enderle D., Berking C., Skog J., Noerholm M. Extracellular vesicles from plasma have higher tumour RNA fraction than platelets. J. Extracell Vesicles. 2020; 9:1741176. 32341768 10.1080/20013078.2020.1741176 PMC7170366

[20] Best M.G. , Sol N., Kooi I., Tannous J., Westerman B.A., Rustenburg F., Schellen P., Verschueren H., Post E., Koster J. et al. RNA-Seq of tumor-educated platelets enables blood-based pan-cancer, multiclass, and molecular pathway cancer diagnostics. Cancer Cell. 2015; 28:666–676. 26525104 10.1016/j.ccell.2015.09.018 PMC4644263

[21] Krug A.K. , Enderle D., Karlovich C., Priewasser T., Bentink S., Spiel A., Brinkmann K., Emenegger J., Grimm D.G., Castellanos-Rizaldos E. et al. Improved EGFR mutation detection using combined exosomal RNA and circulating tumor DNA in NSCLC patient plasma. Ann. Oncol. 2018; 29:700–706. 29216356 10.1093/annonc/mdx765 PMC5889041

[22] Vitale S.R. , Helmijr J.A., Gerritsen M., Coban H., van Dessel L.F., Beije N., van der Vlugt-Daane M., Vigneri P., Sieuwerts A.M., Dits N. et al. Detection of tumor-derived extracellular vesicles in plasma from patients with solid cancer. BMC Cancer. 2021; 21:315. 33761899 10.1186/s12885-021-08007-z PMC7992353

[23] Wang L. , Yekula A., Muralidharan K., Small J.L., Rosh Z.S., Kang K.M., Carter B.S., Balaj L. Novel gene fusions in glioblastoma tumor tissue and matched patient plasma. Cancers (Basel). 2020; 12:1219. 32414213 10.3390/cancers12051219 PMC7281415

[24] Khandelwal G. , Girotti M.R., Smowton C., Taylor S., Wirth C., Dynowski M., Frese K.K., Brady G., Dive C., Marais R. et al. Next-generation sequencing analysis and algorithms for PDX and CDX models. Mol. Cancer Res. 2017; 15:1012–1016. 28442585 10.1158/1541-7786.MCR-16-0431

[25] Callari M. , Batra A.S., Batra R.N., Sammut S.J., Greenwood W., Clifford H., Hercus C., Chin S.F., Bruna A., Rueda O.M. et al. Computational approach to discriminate human and mouse sequences in patient-derived tumour xenografts. BMC Genomics. 2018; 19:19. 29304755 10.1186/s12864-017-4414-y PMC5755132

[26] Kluin R.J.C. , Kemper K., Kuilman T., de Ruiter J.R., Iyer V., Forment J.v., Cornelissen-Steijger P., de Rink I., ter Brugge P., Song J.Y. et al. XenofilteR: Computational deconvolution of mouse and human reads in tumor xenograft sequence data. BMC Bioinform. 2018; 19:366. 10.1186/s12859-018-2353-5 PMC6172735 30286710

[27] Everaert C. , Helsmoortel H., Decock A., Hulstaert E., van Paemel R., Verniers K., Nuytens J., Anckaert J., Nijs N., Tulkens J. et al. Performance assessment of total RNA sequencing of human biofluids and extracellular vesicles. Sci. Rep. 2019; 9:17574. 31772251 10.1038/s41598-019-53892-x PMC6879519

[28] Harenza J.L. , Diamond M.A., Adams R.N., Song M.M., Davidson H.L., Hart L.S., Dent M.H., Fortina P., Reynolds C.P., Maris J.M. Data Descriptor: Transcriptomic profiling of 39 commonly-used neuroblastoma cell lines. Sci. Data. 2017; 4:170033. 28350380 10.1038/sdata.2017.33 PMC5369315

[29] Deveson I.W. , Chen W.Y., Wong T., Hardwick S.A., Andersen S.B., Nielsen L.K., Mattick J.S., Mercer T.R. Representing genetic variation with synthetic DNA standards. Nat. Methods. 2016; 13:784–791. 27502217 10.1038/nmeth.3957

[30] Hulstaert E. , Decock A., Morlion A., Everaert C., Verniers K., Nuytens J., Nijs N., Schroth G.P., Kuersten S., Gross S.M. et al. Messenger RNA capture sequencing of extracellular RNA from human biofluids using a comprehensive set of spike-in controls. STAR Protoc. 2021; 2:100475. 33937877 10.1016/j.xpro.2021.100475 PMC8076706

[31] Ahdesmaki M.J. Improved PDX and CDX data processing-letter. Mol. Cancer Res. 2018; 16:1813. 30385665 10.1158/1541-7786.MCR-18-0534

[32] Ahdesmäki M.J. , Gray S.R., Johnson J.H., Lai Z. Disambiguate: an open-source application for disambiguating two species in next generation sequencing data from grafted samples. F1000Res. 2017; 5:2741. 10.12688/f1000research.10082.1 PMC5130069 27990269

[33] Jardim-Perassi B.V. , Alexandre P.A., Sonehara N.M., de Paula-Junior R., Reis Júnior O., Fukumasu H., Chammas R., Coutinho L.L., Zuccari D.A.P.deC. RNA-Seq transcriptome analysis shows anti-tumor actions of melatonin in a breast cancer xenograft model. Sci. Rep. 2019; 9:966. 30700756 10.1038/s41598-018-37413-w PMC6353949

[34] Anckaert J. , Avila Cobos F., Decock A., Deleu J., de Wever O., de Wilde J., Dhondt B., D’huyvetter T., Everaert C., Fierro C. et al. Performance of RNA purification kits and blood collection tubes in the Extracellular RNA Quality Control (exRNAQC) study. 2021; bioRxiv doi:11 May 2021, preprint: not peer reviewed. 10.1101/2021.05.11.442610.

[35] Godsey J.H. , Silvestro A., Barrett J.C., Bramlett K., Chudova D., Deras I., Dickey J., Hicks J., Johann D.J., Leary R. et al. Generic protocols for the analytical validation of next-generation sequencing-based ctDNA Assays: a Joint Consensus Recommendation of the BloodPAC’s Analytical Variables Working Group. Clin. Chem. 2020; 66:1156–1166. 32870995 10.1093/clinchem/hvaa164 PMC7462123

[36] Brand A. , Allen L., Altman M., Hlava M., Scott J. Beyond authorship: attribution, contribution, collaboration, and credit. Learned Publish. 2015; 28:151–155.

