# Supplementary material for: Whole transcriptome profiling of liquid biopsies from tumour xenografted mouse models enables specific monitoring of tumour-derived extracellular RNA
Source: NAR Cancer. 2022 Nov 28;4(4):zcac037. doi: 10.1093/narcan/zcac037 (PMC9703587; doi:10.1093/narcan/zcac037)
Supplement: zcac037_Supplemental_Files [file zcac037_supplemental_files.zip › Supplemental.pdf]

Whole transcriptome profiling of liquid biopsies from tumour xenografted mouse models enables specific monitoring of tumour-derived extracellular RNA

Vanessa Vermeirssen[1,2,3,4†*], Jill Deleu[2,3†], Annelien Morlion[2,3], Celine Everaert[2,3], Jilke De Wilde[3,5], Jasper Anckaert[2,3], Kaat Durinck[3,6], Justine Nuytens[2,3], Muhammad Rishfi[3,6], Frank Speleman[3,6], Hanne Van Droogenbroeck[2,3], Kimberly Verniers[2,3], Maria Francesca Baietti[7,8], Maarten Albersen[9], Eleonora Leucci[7,8], Edward Post[10,11], Myron G. Best[10,11], Tom Van Maerken[2,3,12], Bram De Wilde[2,3,13], Jo Vandesompele[2,3,$] and Anneleen Decock[2,3,$]

[1]Department of Biomedical Molecular Biology, Ghent University, Ghent, Belgium
[2]OncoRNALab, Cancer Research Institute Ghent (CRIG), Ghent, Belgium
[3]Department of Biomolecular Medicine, Ghent University, Ghent, Belgium
[4]CBIGR, Cancer Research Institute Ghent (CRIG), Ghent, Belgium
[5]Department of Pathology, Ghent University Hospital, Ghent, Belgium
[6]PPOL, Cancer Research Institute Ghent (CRIG), Ghent, Belgium
[7]Laboratory for RNA Cancer Biology, Department of Oncology, KU Leuven, Leuven, Belgium
[8]TRACE, Leuven Cancer Institute, KU Leuven, Belgium
[9]Laboratory of Experimental Urology, Department of Development and Regeneration, KU Leuven, Department of Urology, University Hospitals Leuven, Leuven, Belgium
[10]Amsterdam UMC Location Vrije Universiteit Amsterdam, Department of Neurosurgery, Boelelaan 1117, Amsterdam, the Netherlands
[11]Cancer Center Amsterdam, Brain Tumor Center and Liquid Biopsy Center, Amsterdam, the Netherlands
[12]Department of Laboratory Medicine, AZ Groeninge, Kortrijk, Belgium
[13]Department of Paediatric Haematology Oncology and Stem Cell Transplantation, Ghent University Hospital, Ghent, Belgium

*To whom correspondence should be addressed. Tel: +329 233 39 45; Email: vanessa.vermeirssen@ugent.be.
[†]The authors wish it to be known that, in their opinion, the first two authors should be regarded as joint First Authors
[$]The authors wish it to be known that, in their opinion, the last two authors should be regarded as joint Last Authors

SUPPLEMENTAL MATERIAL AND METHODS

For validation purposes, the exRNAxeno pipeline was applied to liquid biopsies from 20 additional PDX or CDX mice. Below we describe the collection, processing and analysis of these samples.

Liquid biopsy collection

In vivo work for the neuroblastoma IMR-32 CDX model (Table 1) was previously described (1). Liquid biopsies were prepared from vehicle-treated animals (n = 7). When mice reached a maximal tumour volume of 2000 mm³ or specific humane endpoints (i.e. a weight gain or loss of more than 20% or clinical signs of significant pain, distress or suffering), mice were sacrificed by cardiac puncture for liquid biopsy collection (blood volume range between 0.8 and 1 ml), followed by cervical dislocation. Liquid biopsies (SSP, DSP and TSP) were collected according to the protocol described in Supplemental Figure 1. The degree of haemolysis of TSP plasma samples was assessed by measuring levels of haemoglobin by spectrophotometric analysis (OD414) using a NanoDrop 1000 Spectrophotometer (Thermo Fisher Scientific, Waltham, MA, USA; Supplemental table 2).

In vivo work for the neuroblastoma (SK-N-BE(2C) (n=3)), melanoma (MEL0002 (n=2), MEL0068 (n=1) and MEL0083 (n=1)), endometrial (EMC0052 (n=2) and EMC0078 (n=1)), penile (PEN0014 (n=1) and PEN0020 (n=1)) and lung (A549 (n=1)) cancer xenograft mice models was done at the TRACE PDX platform (KU Leuven, UZ Leuven, Belgium, approved by the local ethical committee for animal experimentation (P164/2019)). To establish the PDX models (https://gbiomed.kuleuven.be/english/research/50488876/54502087/Trace/PDX-repository; Table 1), tumour fragments freshly isolated from patients were implanted interscapular or in the flank of female immunodeficient nude mice (NMRI-Foxn1^{nu} strain, Taconic Biosciences, Rensselaer, NY, USA or NSG, Charles River, Wilmington, MA, USA). Tumours were propagated in at least 3 generations of mice and characterized by histology and SNP fingerprinting to confirm genealogy before liquid biopsy collection. All patients of whom tumour material was collected, provided informed consent prior to study participation, approved by the Ethical Committee UZ Leuven (S63799, S61605 and S66742). To establish the SK-N-BE(2C) and A549 CDX models, female NMRI-Foxn1^{nu} mice were subcutaneously xenografted in the dorsal flank with 2 x 10^6 cells, in 30 µl RPMI 1640 medium (Thermo Fisher Scientific, Waltham, MA, USA), suspended in 70 µl Matrigel matrix (Corning, Bedford, MA, UK) at the age of 13 weeks. When PDX or CDX mice reached at least 1000 mm³ or specific humane endpoints (i.e. a weight loss of more than 20% or clinical signs of significant pain, distress or suffering), mice were sacrificed by cardiac puncture for liquid biopsy collection (blood volume range between 0.5 and 1.1 ml) followed by cervical dislocation. Liquid biopsies (SSP, DSP and TSP) were collected according to the protocol described in Supplemental Figure 1. The degree of haemolysis of all plasma samples was assessed by measuring levels of haemoglobin by spectrophotometric analysis (OD414) using a NanoDrop 1000 Spectrophotometer (Thermo Fisher Scientific, Waltham, MA, USA; Supplemental table 2).

RNA isolation, spike-in RNA addition and DNase treatment

Extracellular RNA from 60 µl plasma (70 µl plasma for IMR-32 CDX mice), was isolated using the miRNeasy Serum/Plasma Kit (Qiagen, Hilden, Germany), according to the manufacturer's manual. During RNA extraction, 2 µl of a 100,000-fold dilution of Sequin spike-in controls (Garvan Institute of Medical Research,

Darlinghurst, NSW, Australia (2)) was added to the lysate. Upon RNA purification, 2 µl of a 70,000-fold dilution of External RNA Control Consortium (ERCC) RNA Spike-in Mix (Thermo Fisher Scientific, Waltham, MA, USA) was added to 12 µl RNA eluate, followed by gDNA removal (3). To this purpose, 1 µl HL-dsDNase (ArcticZymes Technologies, Tromsø, Norway) and 1.4 µl Heat & Run 10X Reaction Buffer (ArcticZymes Technologies, Tromsø, Norway) were added to the eluates, and RNA samples were incubated for 10 min at 37 °C, followed by 5 min at 58 °C.

Total RNA library preparation and sequencing

Total RNA libraries were prepared starting from 8 µl DNase-treated RNA using the SMARTer Stranded Total RNA-Seq Kit v2 - Pico Input Mammalian (Takara Bio, CA, USA), according to the manufacturer's manual with minor modifications (4). Briefly, prior to first strand cDNA synthesis, RNA from liquid biopsies was fragmented for 2 min at 94 °C. During final library amplification, 16 PCR cycles were performed on the liquid biopsy samples. The final clean-up was repeated, since an excessive number of products < 200 bp in size was observed on Fragment Analyzer data (data not shown, Agilent Technologies, Santa Clara, CA, USA). Fragment sizes were determined using Fragment Analyzer software for smear analysis in the 200 bp to 1000 bp range. Library quantification was performed using the KAPA Library quantification Kit (Kapa Biosystems, Wilmington, MA, USA) and libraries were pooled equimolarly. The final pool was quantified using Qubit, and 0.61 nM was loaded on a Novaseq instrument (Novaseq SP Kit v1.5, 200 cycles), with 2% PhiX. Raw sequencing data is available in the European Genome-Phenome archive (EGAS00001006582).

Preprocessing of RNA sequencing data

Sequencing reads of liquid biopsy and tumour samples were preprocessed by FastQC (v.0.11.8) for quality control and trimmed by Cutadapt (v.1.18) for low quality bases at the 3' end of each read (Q30), for 3 nucleotides from the 5' end of the second read (due to the template switching adapter) and for the HT-TruSeq adapter sequences. Reads shorter than 35 bp were filtered out. Next, duplicated reads were removed with Clumpify (BBMap v.38.26) within clumps based on 60 bp trimmed reads and using default parameters, except for 20 passes. Subsequently, reads were once more analysed by FastQC for quality control.

exRNAxeno computational framework for combined mapping of RNA sequencing data

Reads were mapped using STAR (v.2.6.0) (specific parameter settings: --outSAMprimaryFlag AllBestScore --outSAMattributes NH HI AS nM NM) to a combined reference genome of human and mouse (combined mapping). The genome index for mapping was built using the Ensembl GRCh38.94 (human) and GRCm38.94 (mouse) DNA primary assembly sequences, containing all chromosomes, the mitochondrial genome and scaffolds, supplemented with ERCC and

Sequin spike sequences and the full ribosomal DNA complete repeating unit (U13369.1, BK000964.3). GTF files were downloaded from Ensembl and adapted in a similar way. In the combined reference genome of mouse and human, mouse chromosomes were labelled with a prefix 'm'. Uniquely mapped reads were selected based on the NH:i:1 tag (SAMtools v.1.8, Pysam). BAM files were further masked by intersectBed for regions where control murine (n = 15) and human (n = 4) liquid biopsies empirically showed misalignment to the other reference genome (SAMtools v.1.8, BEDtools v.2.27.1, BEDOPS v.2.4.32). Further quality control on the filtered BAM files was done using MultiQC (v.1.7), SAMtools (v.1.8), RseQC (v.2.6.4) and BEDTools (v.2.27.1). Finally, read counts of name-sorted BAM files were generated by HTSeq-count (v.0.11.0) (specific parameter settings: -s reverse –secondary-alignments=ignore –supplementary-alignments=ignore) using appropriate GTF files. Further processing was done with R (v.4.0.3) making use of tidyverse (v.1.3.1). The exRNAxeno combined is available through GitHub (https://github.com/CBIGR/exRNAxeno).

Table 1. Information on xenograft models.

Xenograft model	Patient diagnosis	Patient age (years)	Patient gender	Primary tumor metastasis	Tumor grade or stage	Tumor characteristics	Host mouse strain	Implantation site	Tumor volume at collection (mm^3)	Passage at collection	Source	Ref
BRC0004 PDX	breast invasive ductal adenocarcinoma	30-35	female	primary tumor	grade 3, pT2N0	triple negative: ER-, PR-, $HER2_{low}$	NMRI-$Foxn1^{nu}$	interscapular	1104-1286	unknown	UZ Leuven, TRACE	(5)
SK-N-BE(2C) CDX	neuroblastoma	0-5	male	metastasis	stage 4	MYCN amplification	NMRI- $Foxn1^{nu}$	flank	1166 - 2243	unknown	ATCC	CRL-2271, ATCC
IMR-32 CDX	neuroblastoma	0-5	male	primary tumor	unknown	MYCN amplification	NU- $Foxn1^{nu}$	flank	1949 - 2271	unknown	UGent, PPOL	(1)
MEL0083 PDX	cutaneous melanoma	unknown	male	metastasis	unknown	NRAS Q61R	NMRI- $Foxn1^{nu}$	interscapular	2756	P5	UZ Leuven, TRACE	(6)
MEL0068 PDX	cutaneous melanoma	65-70	female	metastasis	unknown	BRAF mutant	NMRI- $Foxn1^{nu}$	interscapular	2043	P5	UZ Leuven, TRACE	(6–8)
MEL0002 PDX	cutaneous melanoma	55-60	male	metastasis	unknown	BRAF mutant	NMRI- $Foxn1^{nu}$	interscapular	1107-1383	P8	UZ Leuven, TRACE	(6–8)
EMC0052 PDX	uterine leiomyosarcoma	55-60	female	metastasis	grade 3	$Vimentin_{low}$, Desmin-, Caldesmon-	NSG	flank	1311-2013	P5	UZ Leuven, TRACE	(9)
EMC0078 PDX	uterine leiomyosarcoma	60-65	female	metastasis	grade 3	Vimentin+, Desmin+, Caldesmon+	NSG	flank	2638	P7	UZ Leuven, TRACE	(9)

Xenograft model	Patient diagnosis	Patient age (years)	Patient gender	Primary tumor metastasis	Tumor grade or stage	Tumor characteristics	Host mouse strain	Implantation site	Tumor volume at collection (mm³)	Passage at collection	Source	Ref
PEN0014 PDX	penile squamous cell carcinoma	75-80	male	metastasis	grade 2	HPV+	NMRI- Foxn1^{nu}	interscapular	2105	P2	UZ Leuven, TRACE	(10)
PEN0020 PDX	penile squamous cell carcinoma	65-70	male	metastasis	grade 2	HPV+	NMRI- Foxn1^{nu}	interscapular	1073	P1	UZ Leuven, TRACE	not published, Albersen, Leucci, *et al.* and (10)
A549 CDX	lung adenocarcinoma	55-60	male	primary tumor	unknown	epithelial-like, KRAS G12S	NMRI- Foxn1^{nu}	flank	1567	unknown	ATCC	CCL-185, ATCC

REFERENCES

1. Nunes,C., Depestel,L., Mus,L., Keller,K.M., Delhaye,L., Louwagie,A., Rishfi,M., Whale,A., Kara,N., Andrews,S.R., *et al.* (2022) RRM2 enhances MYCN-driven neuroblastoma formation and acts as a synergistic target with CHK1 inhibition. *Sci. Adv.*, **8**, eabn1382.

2. Deveson,I.W., Chen,W.Y., Wong,T., Hardwick,S.A., Andersen,S.B., Nielsen,L.K., Mattick,J.S. and Mercer,T.R. (2016) Representing genetic variation with synthetic DNA standards. *Nat. Methods*, **13**, 784–791.

3. Hulstaert,E., Decock,A., Morlion,A., Everaert,C., Verniers,K., Nuytens,J., Nijs,N., Schroth,G.P., Kuersten,S., Gross,S.M., *et al.* (2021) Messenger RNA capture sequencing of extracellular RNA from human biofluids using a comprehensive set of spike-in controls. *STAR Protoc.*, **2**, 100475.

4. Everaert,C., Helsmoortel,H., Decock,A., Hulstaert,E., Van Paemel,R., Verniers,K., Nuytens,J., Anckaert,J., Nijs,N., Tulkens,J., *et al.* (2019) Performance assessment of total RNA sequencing of human biofluids and extracellular vesicles. *Sci. Rep.*, **9**, 1–16.

5. Moens,S., Zhao,P., Baietti,M.F., Marinelli,O., Van Haver,D., Impens,F., Floris,G., Marangoni,E., Neven,P., Annibali,D., *et al.* (2021) The mitotic checkpoint is a targetable vulnerability of carboplatin-resistant triple negative breast cancers. *Sci. Rep.*, **11**, 1–13.

6. Vendramin,R., Katopodi,V., Cinque,S., Konnova,A., Knezevic,Z., Adnane,S., Verheyden,Y., Karras,P., Demesmaeker,E., Bosisio,F.M., *et al.* (2021) Activation of the integrated stress response confers vulnerability to mitoribosome-targeting antibiotics in melanoma. *J. Exp. Med.*, **218**.

7. Leucci,E., Vendramin,R., Spinazzi,M., Laurette,P., Fiers,M., Wouters,J., Radaelli,E., Eyckerman,S., Leonelli,C., Vanderheyden,K., *et al.* (2016) Melanoma addiction to the long non-coding RNA SAMMSON. *Nature*, **531**, 518–522.

8. Rambow,F., Rogiers,A., Marin-Bejar,O., Aibar,S., Femel,J., Dewaele,M., Karras,P., Brown,D., Chang,Y.H., Debiec-Rychter,M., *et al.* (2018) Toward Minimal Residual Disease-Directed Therapy in Melanoma. *Cell*, **174**, 843-855.e19.

9. Cuppens,T., Depreeuw,J., Annibali,D., Thomas,D., Hermans,E., Gommé,E., Trinh,X.B., Debruyne,D., Moerman,P., Lambrechts,D., *et al.* (2017) Establishment and characterization of uterine sarcoma and carcinosarcoma patient-derived xenograft models. *Gynecol. Oncol.*, **146**, 538–545.

10. Elst,L., Van Rompuy,A.S., Roussel,E., Spans,L., Vanden Bempt,I., Necchi,A., Ross,J., Jacob,J.M., Baietti,M.F., Leucci,E., *et al.* (2022) Establishment and Characterization of Advanced Penile Cancer Patient-derived

Tumor Xenografts: Paving the Way for Personalized Treatments. *Eur. Urol. Focus*, 10.1016/j.euf.2022.04.012.

Supplemental figure 1

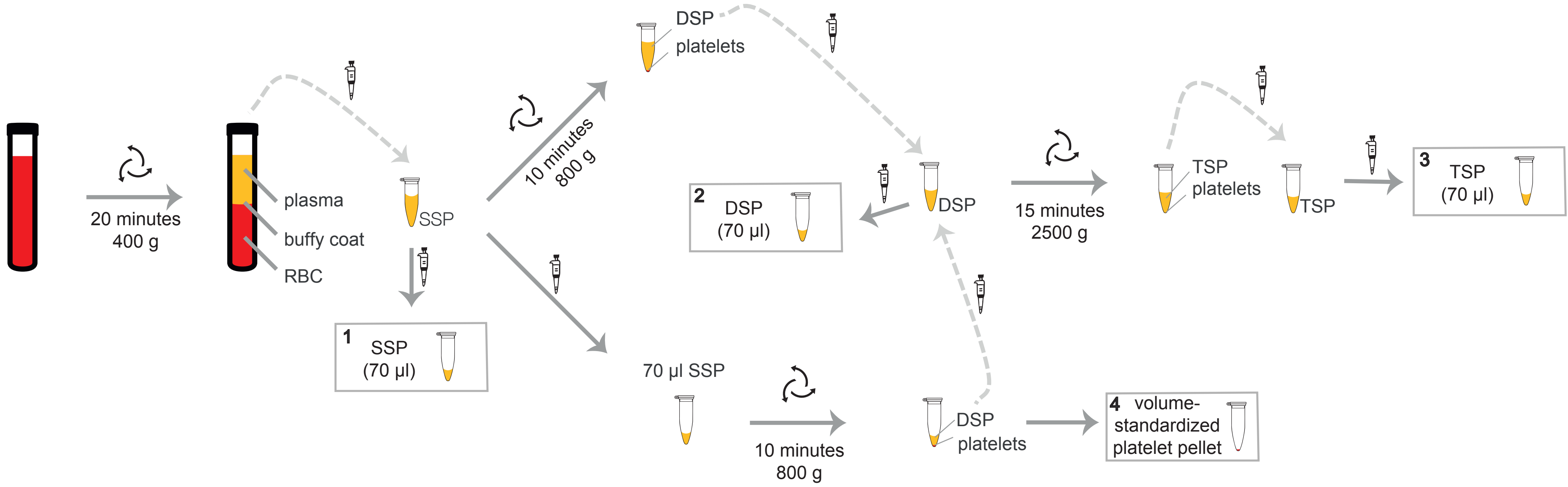

Supplemental figure 1. Liquid biopsy preparation protocol for the non-tumour bearing control mice and SK-N-BE(2C) CDX mice. From each animal, single spun (SSP, box 1), double spun (DSP, box 2), and triple spun (TSP, box 3) plasma, and a volume standardized platelet pellet originating from 70 µl SSP (box 4), were prepared by means of three sequential centrifugation steps. RBC: red blood cells.

Supplemental figure 2

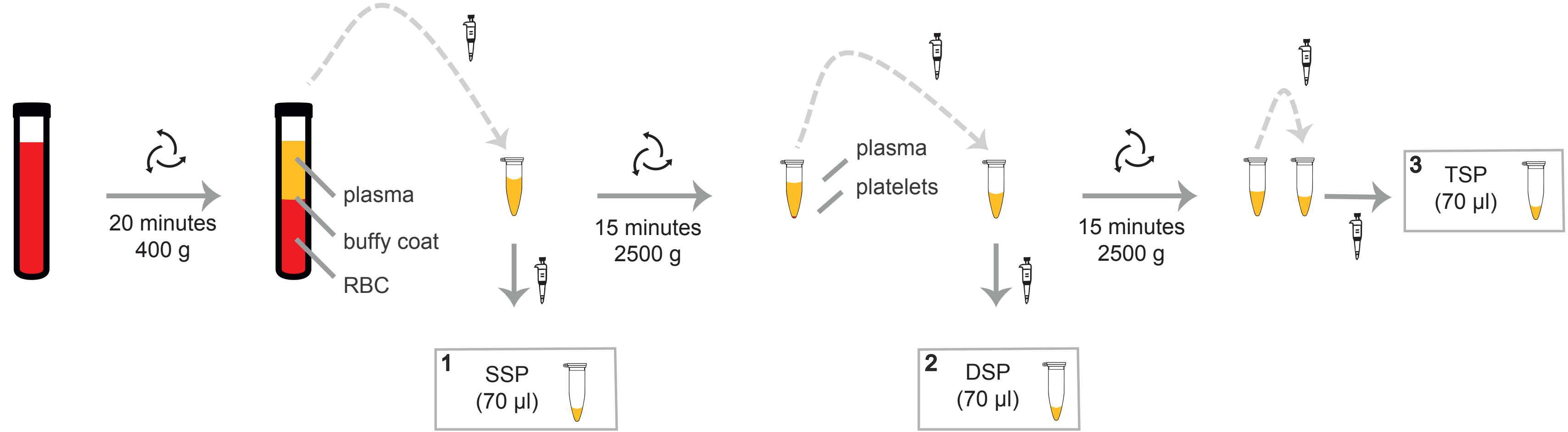

Supplemental figure 2. Liquid biopsy preparation protocol for the BRC0004 PDX mice. From each animal, single spun (SSP, box 1), double spun (DSP, box 2), and triple spun (TSP, box 3) plasma were prepared by means of three sequential centrifugation steps. RBC: red blood cells.

Supplemental figure 3

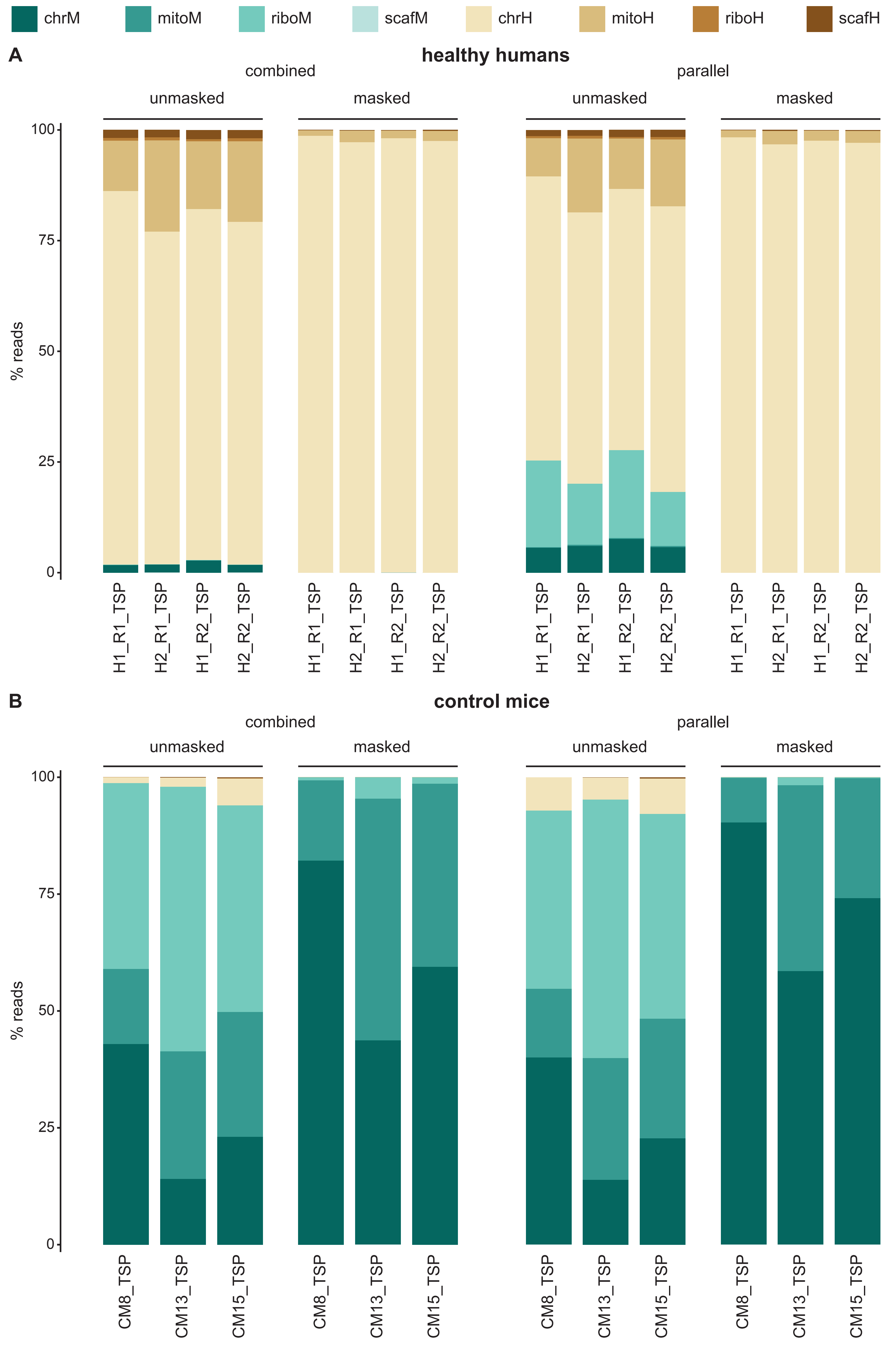

Supplemental figure 3. In the human and non-tumour bearing control murine plasma samples, the masking step in the processing pipelines removes the initially identified genomic regions consisting of misaligned reads. Shown are the relative percentages of total reads derived from human and murine nuclear RNA (chrH and chrM), mitochrondial RNA (mitoH and mitoM), ribosomal RNA (riboH and riboM) and scaffold RNA (scafH and scafM) for both human (A) and non-tumour bearing control murine (B) plasma samples in the combined and parallel mapping, before and after empirical masking of misaligned reads. CM: control mouse; H: human; R: replicate; TSP: triple spun plasma.

Supplemental figure 4

A

SK-N-BE(2C) CDX	murine genes	human genes
platelets	288	28
SSP	225	0
DSP	300	12
TSP	112	0

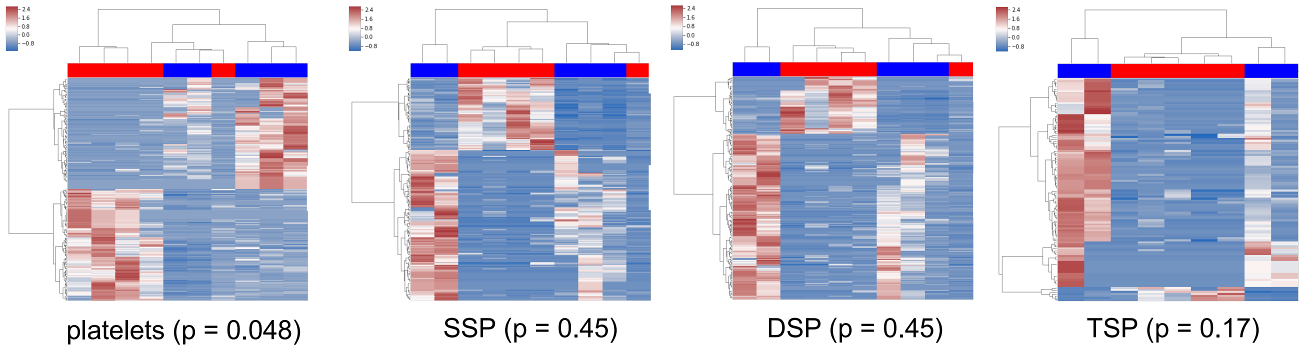

B

BRC0004 PDX	murine genes	human genes
SSP	436	142
DSP	1108	634
TSP	309	156

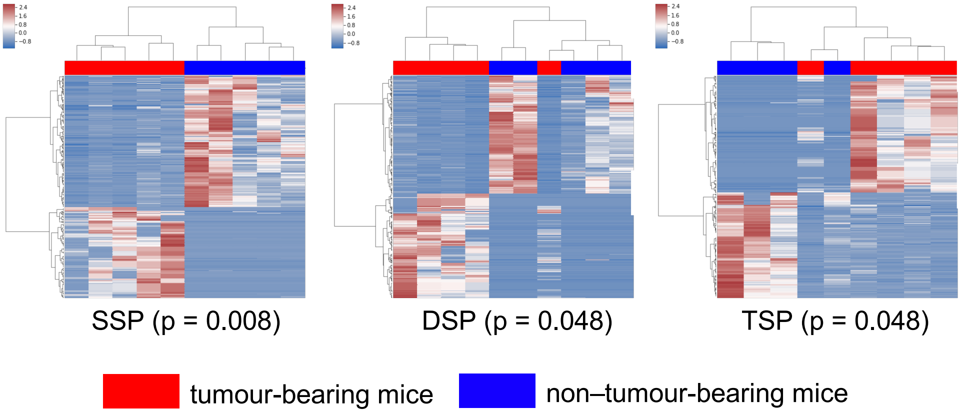

Supplemental figure 4. Tumour-bearing mice contain differential exRNA abundance profiles as compared to non-tumour bearing mice. Number of differential abundant murine and human genes (ANOVA's p-value threshold < 0.05) and heatmaps of unsupervised clustering of platelets, single-spun plasma (SSP), double-spun plasma (DSP) and triple-spun plasma (TSP) from the SK-N-BE(2C) CDX model (A) and BRC0004 PDX model (B). P-values indicate statistical significance of column clustering.

Supplemental figure 5

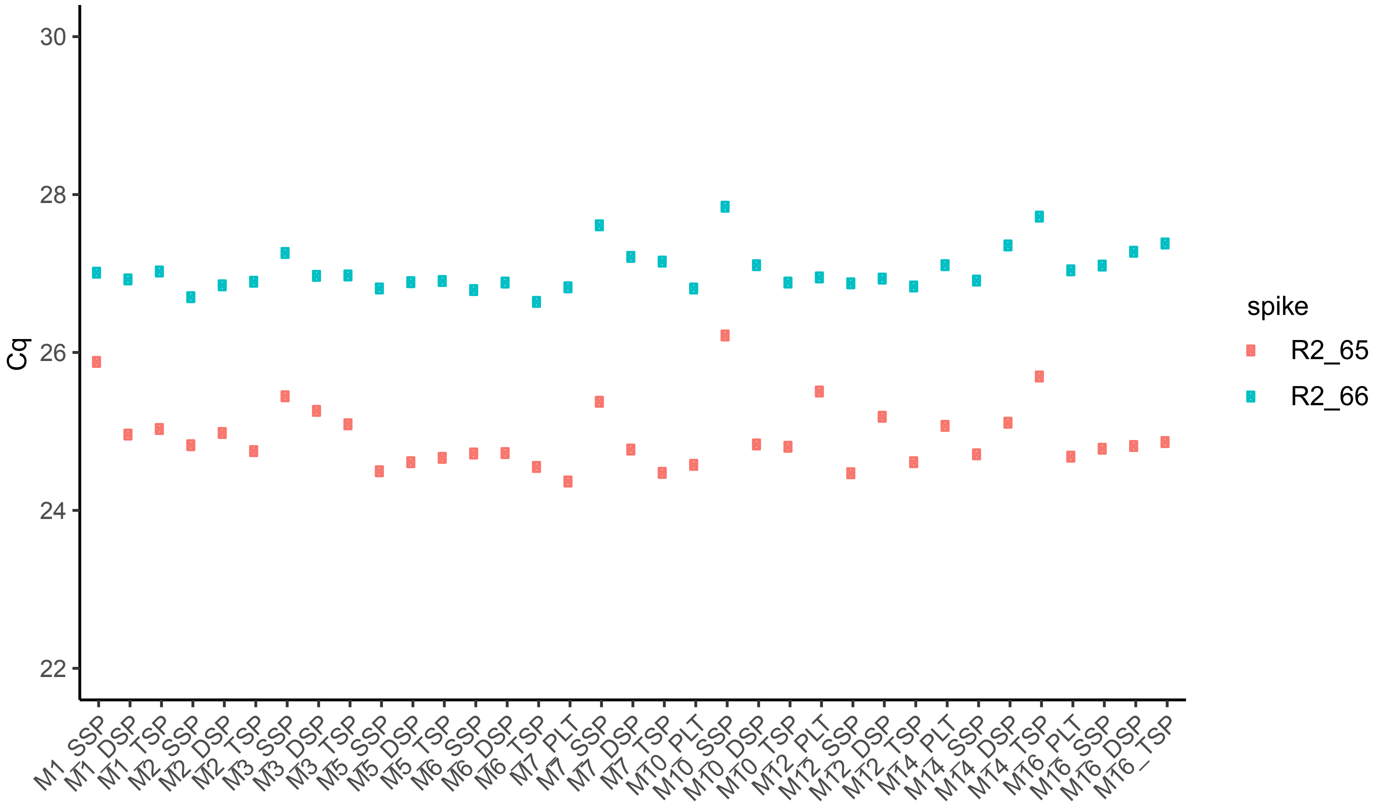

Supplemental figure 5. RT-qPCR analysis for Sequin spikes (R2_65 and R2_66), shows that exRNA purification is reproducible and Sequin spikes can be used for exRNA quantification. The presence of R2_65 and R2_66 Sequin spike-ins in the extracted RNA of all liquid biopsies of the BRC0004 PDX model (M1, M2, M3, M5, M6) and SK-N-BE(2C) CDX model (M7, M10, M12, M14, M16), was confirmed by means of RT-qPCR analysis. DSP: double spun plasma; SSP: single spun plasma; TSP: triple spun plasma

Supplemental figure 6

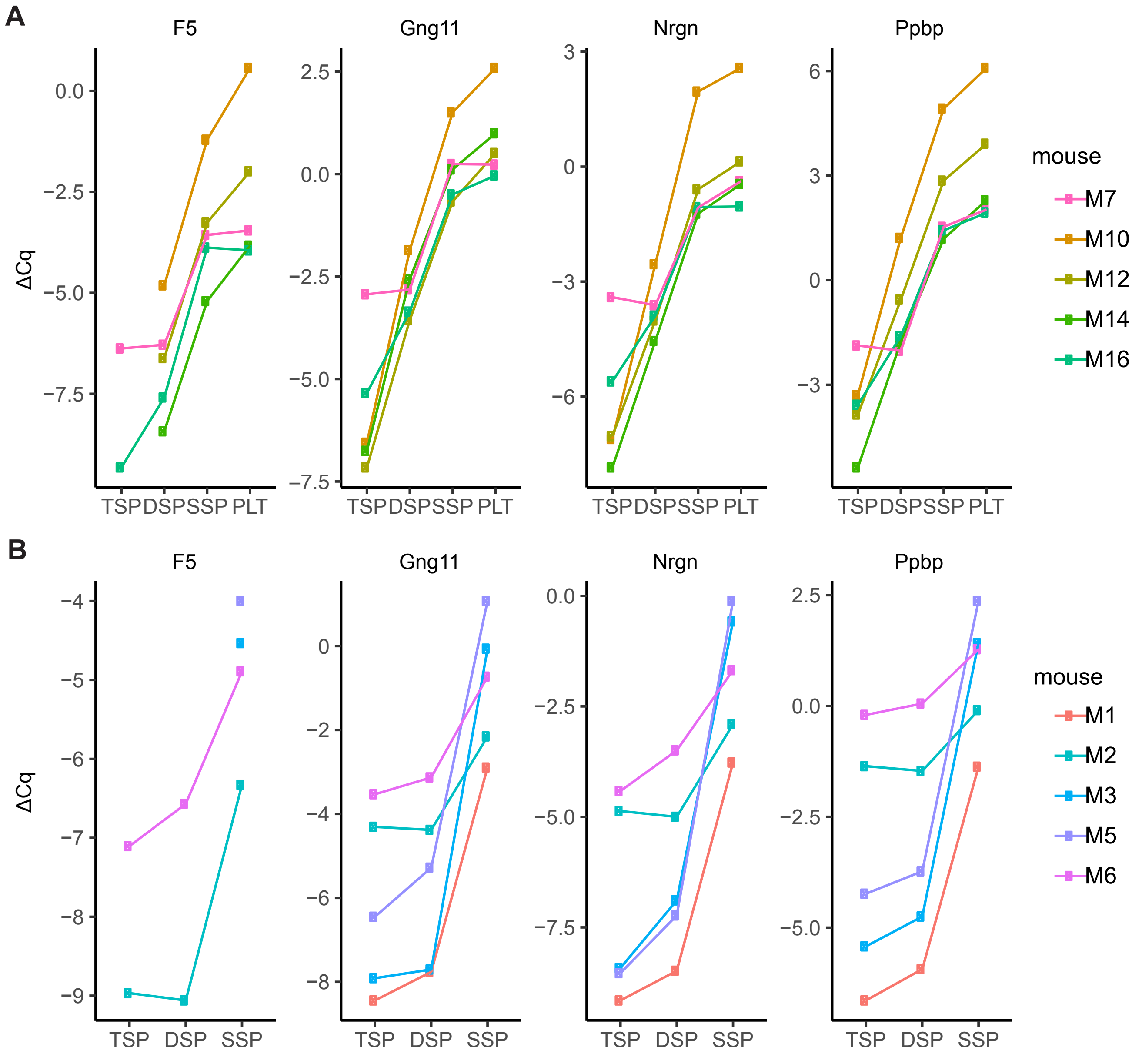

Supplemental figure 6. RT-qPCR analysis for platelet markers confirms a gradual decrease in platelet concentration upon successive centrifugation steps. The ΔCq is obtained by diminishing the mean Cq of R2_65 and R2_66 spikes of each sample by the Cq of each platelet gene of the respective sample. The ΔCq per platelet gene (F5, Gng11, Nrgn and Ppbp) in the different liquid biopsies from the SK-N-BE(2C) CDX model (A) and the BRC0004 PDX model (B) is shown. DSP: double spun plasma; PLT: platelets, SSP: single spun plasma; TSP: triple spun plasma.

Supplemental figure 7

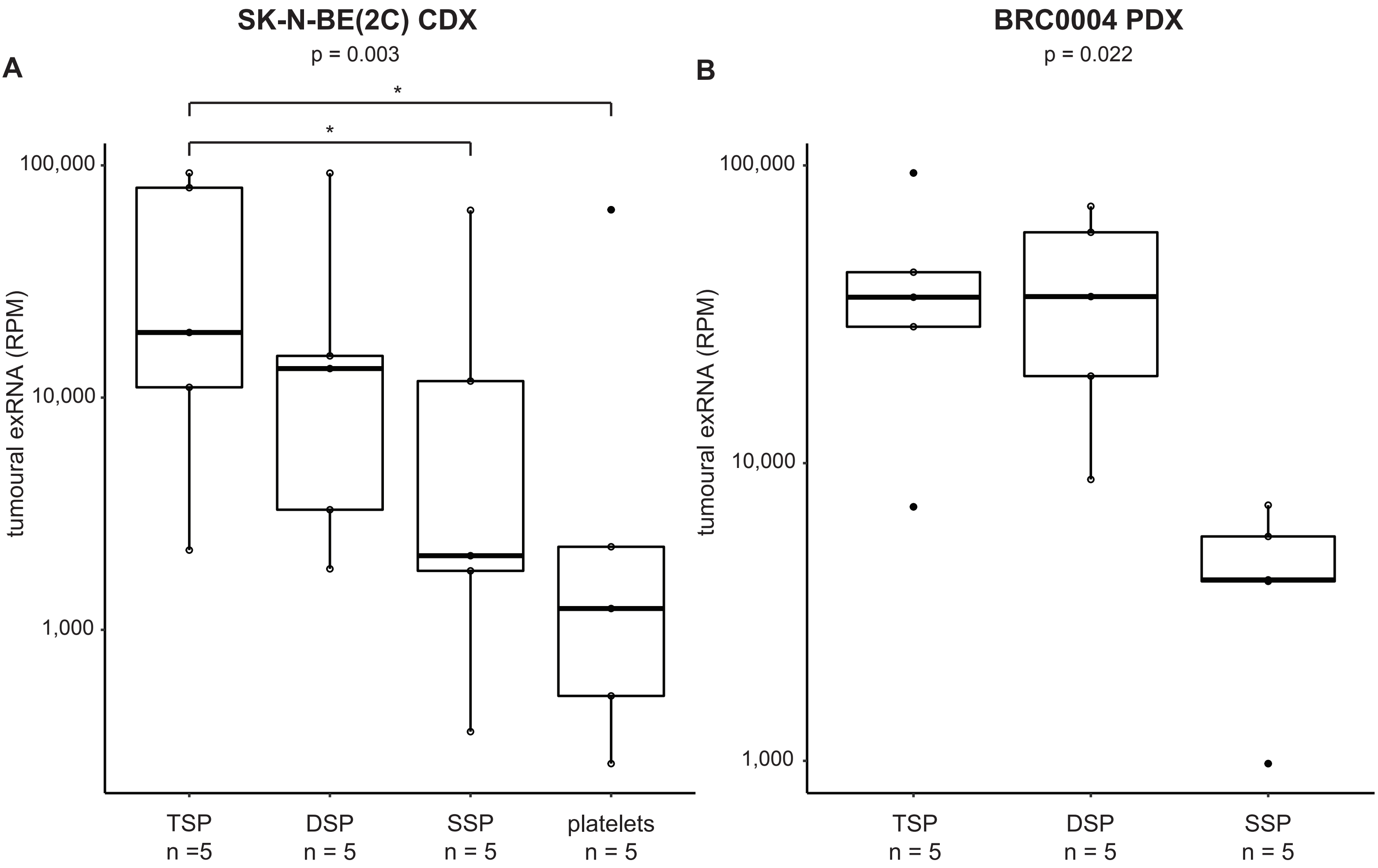

Supplemental figure 7. The relative amount of tumoural exRNA (RPM) is inversely proportional to the platelet plasma level. Shown is the amount of exRNA (RPM, mapped reads per million) present in the different liquid biopsies from the CDX (A; Friedman chi-squared test = 13.56, df = 3, p = 0.003; post-hoc test Nemenyi, p = 0.044 (*)) and PDX (B; Friedman chi-squared test = 7.6, df = 2, p = 0.022) mice. DSP: double spun plasma; SSP: single spun plasma; TSP: triple spun plasma.

Supplemental figure 8

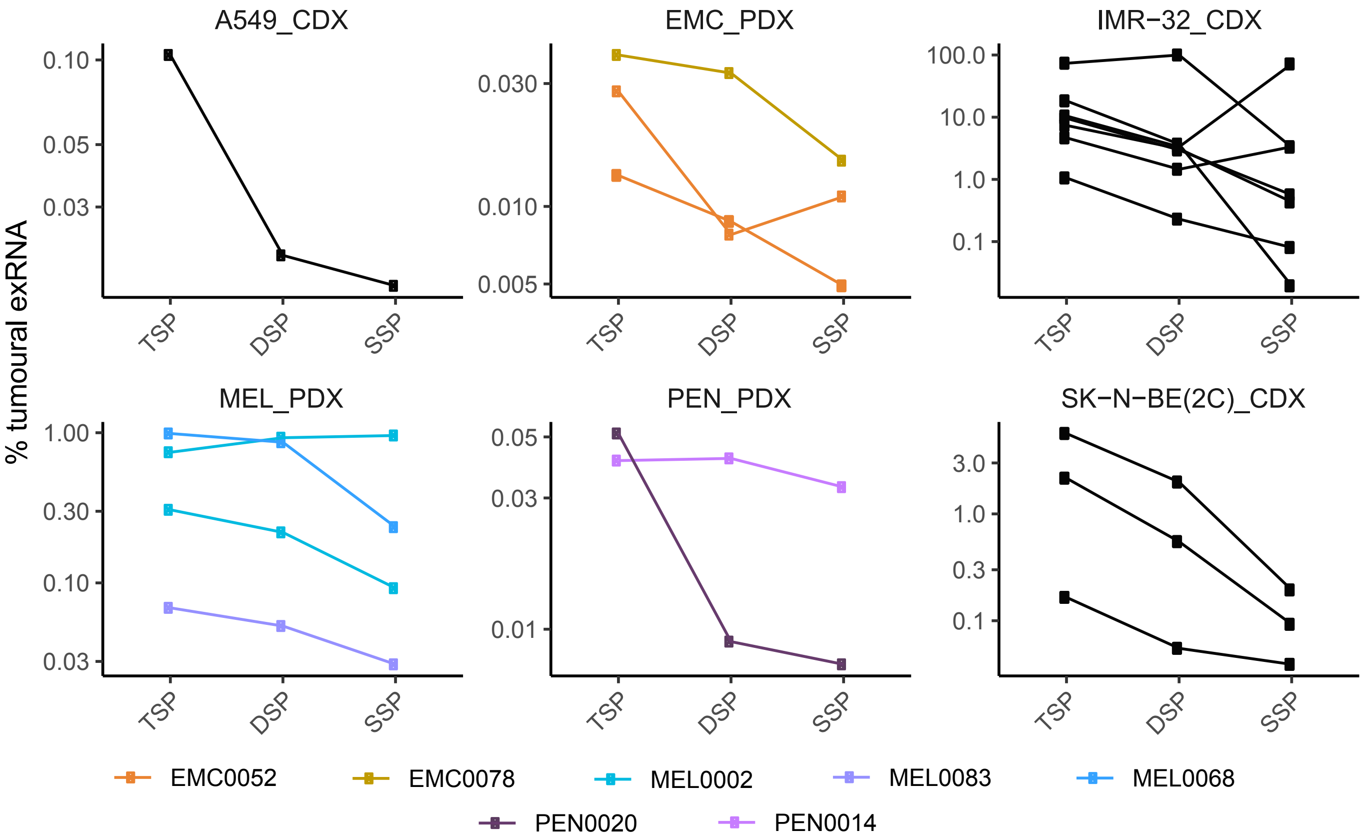

Supplemental figure 8. The tumoural exRNA percentage is inversely proportional to the platelet plasma level for the validation cohort. Shown are the percentages of tumoural exRNA present in the different liquid biopsies from the validation cohort mice, depicted per tumour type (A549: lung cancer, EMC: endometrial cancer, IMR-32: neuroblastoma, MEL: melanoma, PEN: penile cancer and SK-N-BE(2C): neuroblastoma). DSP: double spun plasma; SSP: single spun plasma; TSP: triple spun plasma.

Supplemental figure 9

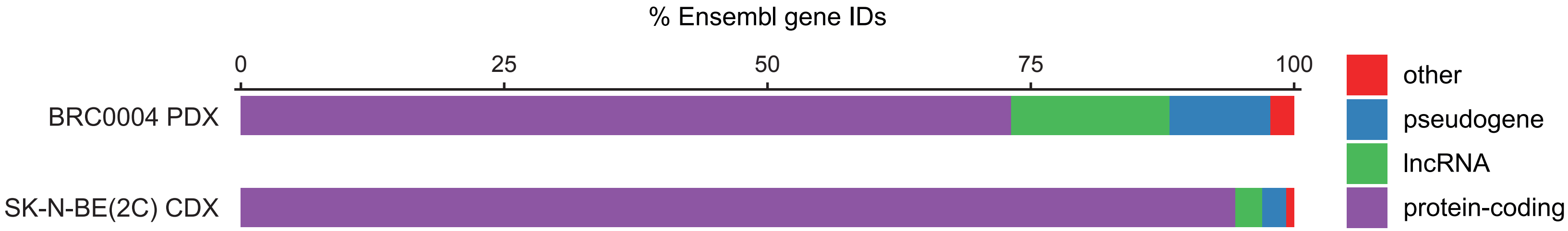

Supplemental figure 9. The majority of the tumour-derived exRNA is protein-coding. For the BRC0004 PDX- and SK-N-BE(2C) CDX-derived liquid biopsies, the percentages of identified human Ensembl IDs annotated as protein-coding, long non-coding RNA (lncRNA), pseudogene or other RNA biotypes are shown.

Supplemental figure 10A

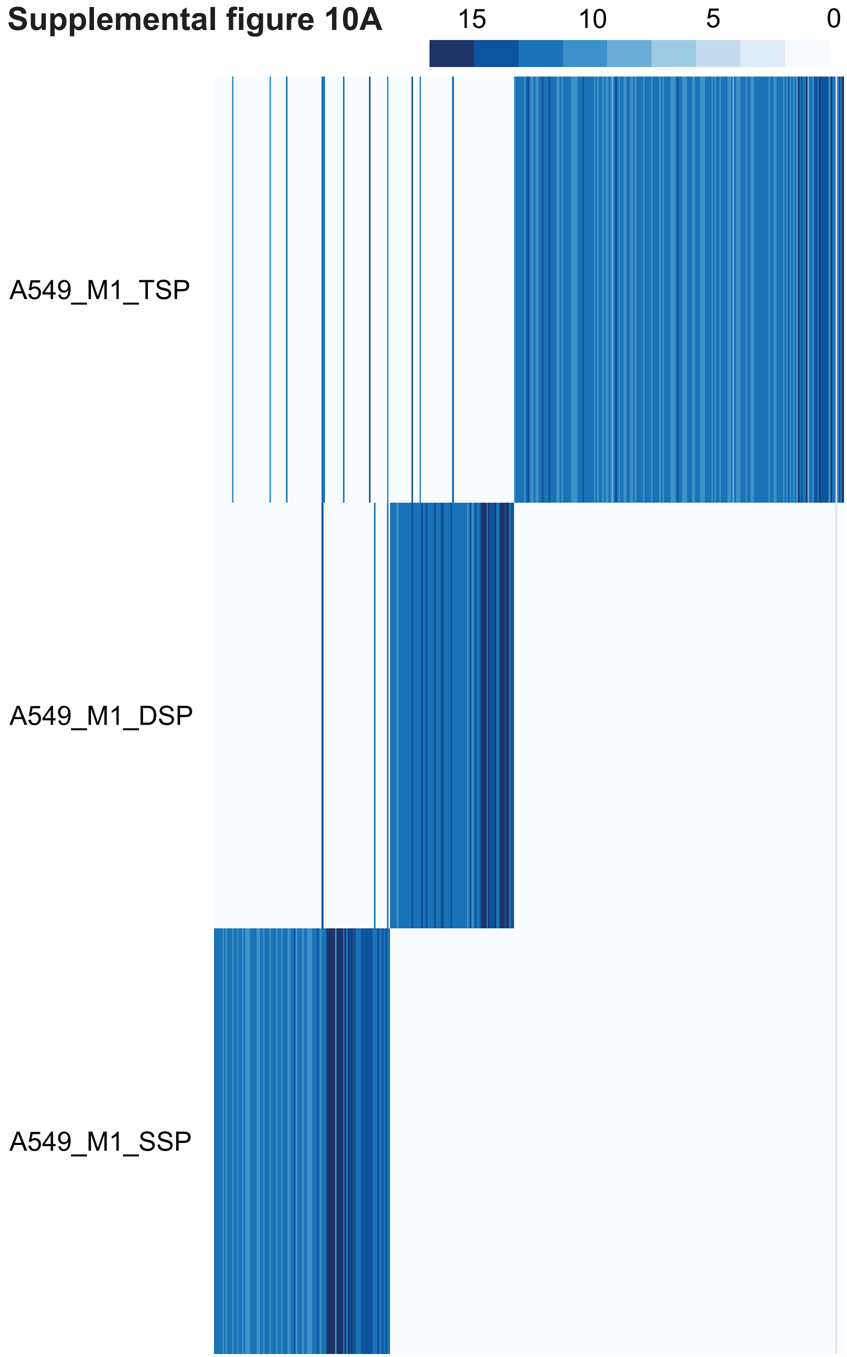

Supplemental figure 10A to F. The circulating tumour gene abundance profile is highly variable across individual mice. Log2(TPM + 1) values of all circulating genes of the validation cohort. DSP: double spun plasma; SSP: single spun plasma; TSP: triple spun plasma.

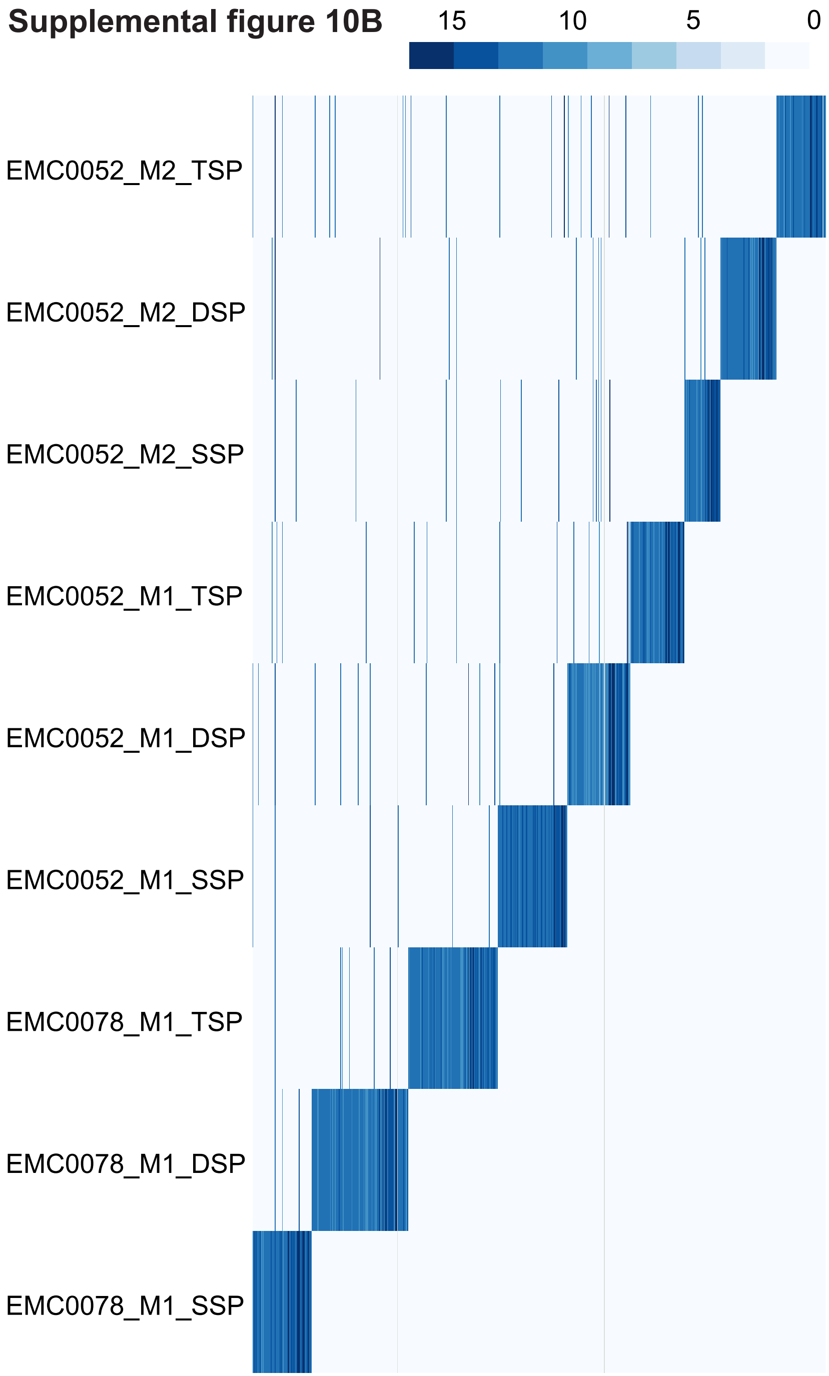

Supplemental figure 10A to F. The circulating tumour gene abundance profile is highly variable across individual mice. Log2(TPM + 1) values of all circulating genes of the validation cohort. DSP: double spun plasma; SSP: single spun plasma; TSP: triple spun plasma.

Supplemental figure 10C

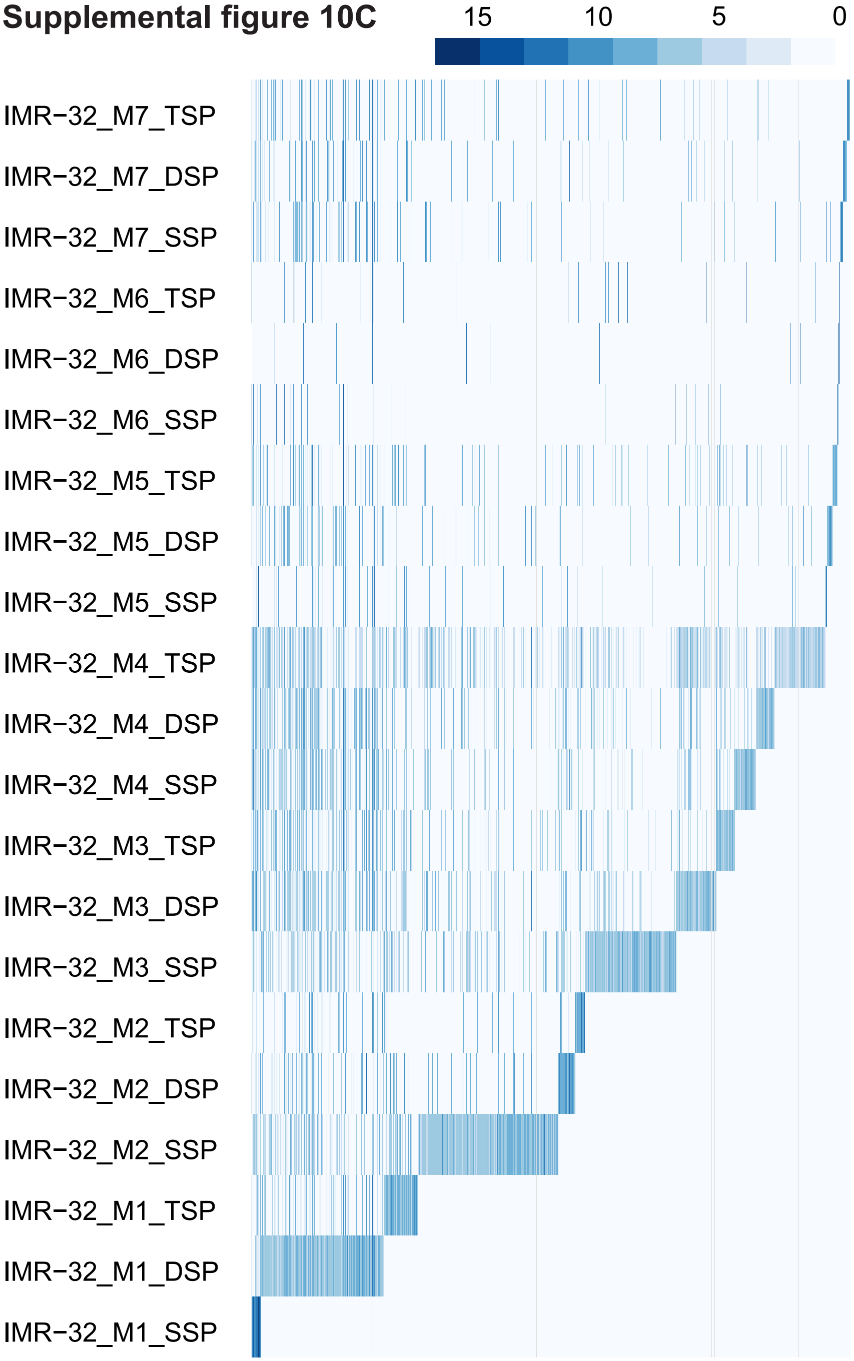

Supplemental figure 10A to F. The circulating tumour gene abundance profile is highly variable across individual mice. Log2(TPM + 1) values of all circulating genes of the validation cohort. DSP: double spun plasma; SSP: single spun plasma; TSP: triple spun plasma.

Supplemental figure 10D

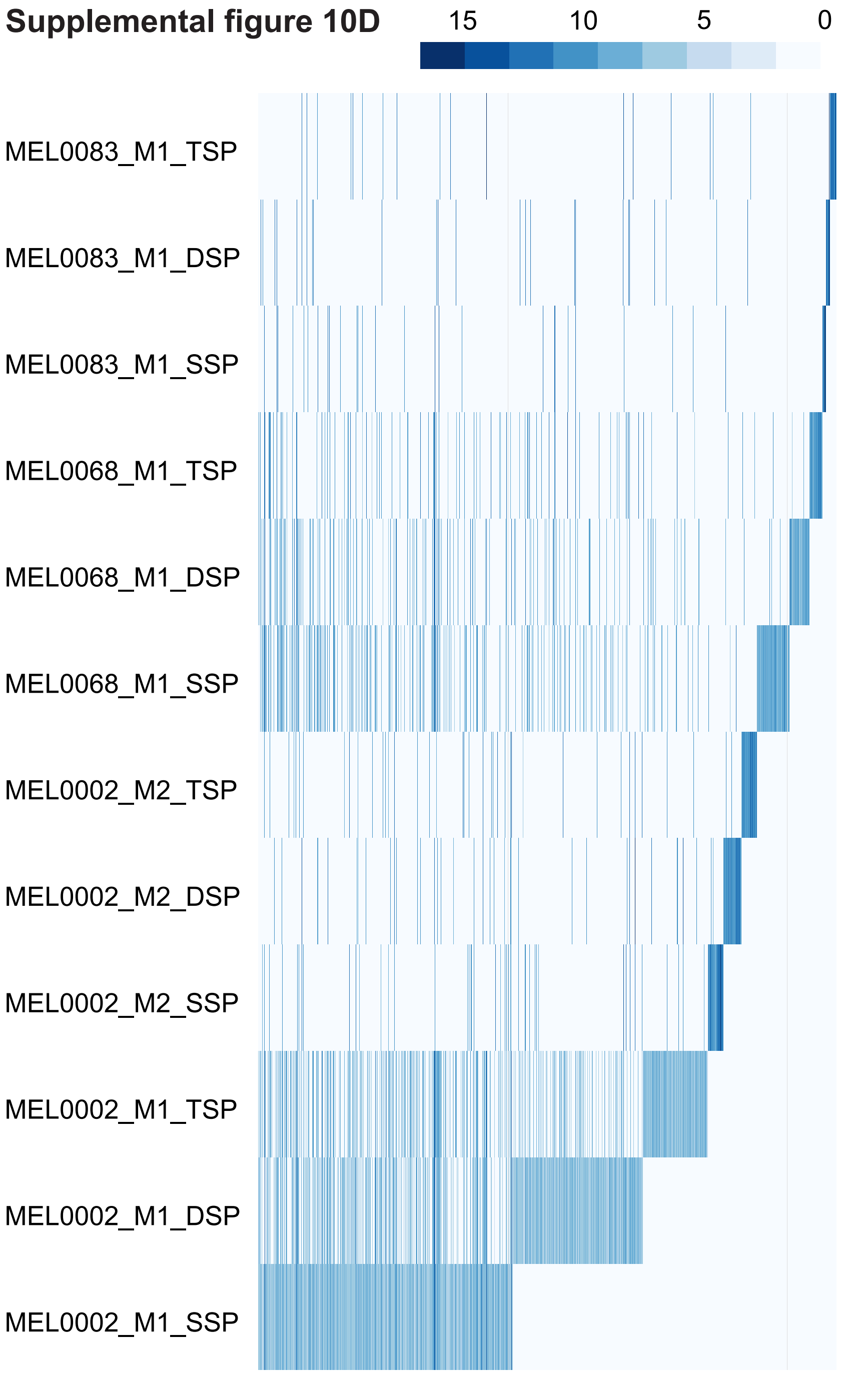

Supplemental figure 10A to F. The circulating tumour gene abundance profile is highly variable across individual mice. Log2(TPM + 1) values of all circulating genes of the validation cohort. DSP: double spun plasma; SSP: single spun plasma; TSP: triple spun plasma.

Supplemental figure 10E

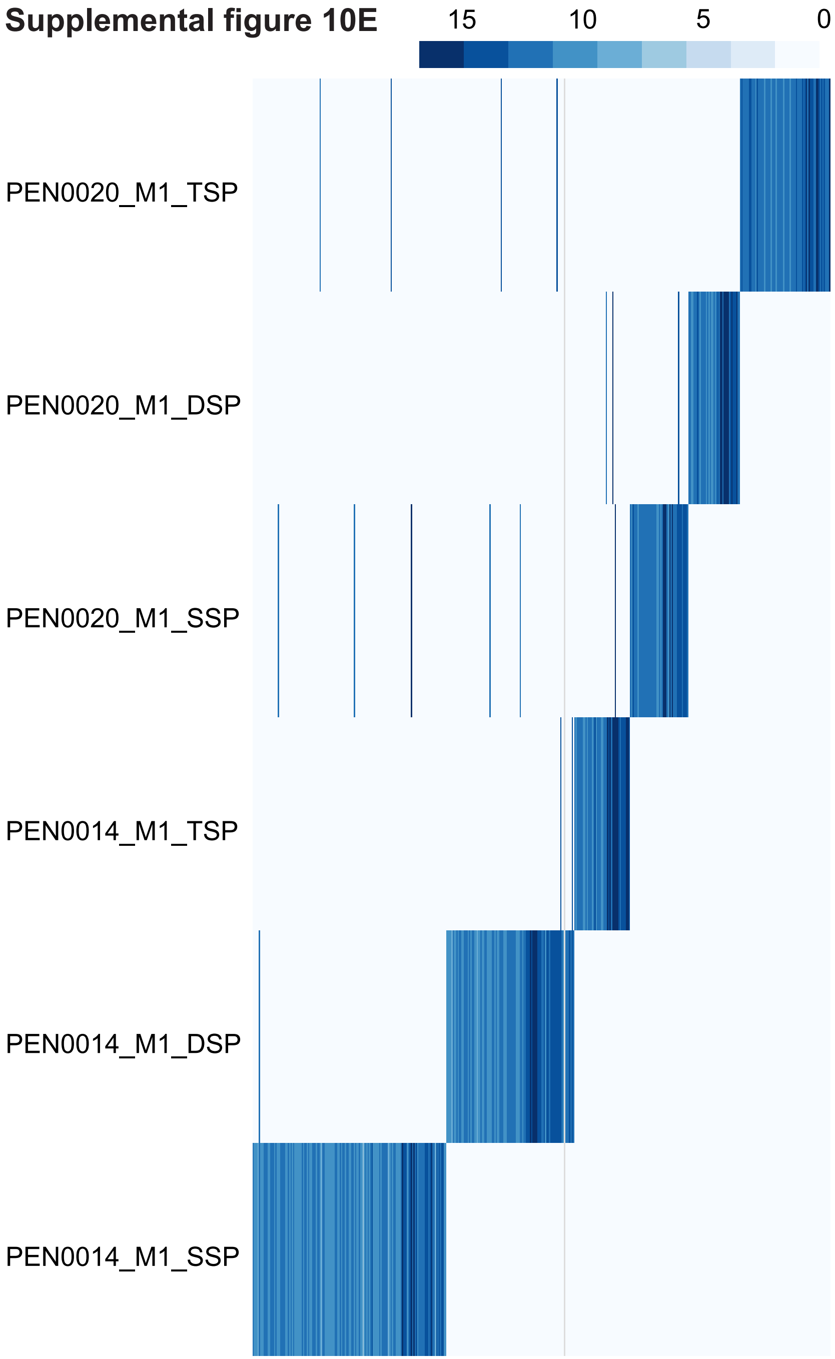

Supplemental figure 10A to F. The circulating tumour gene abundance profile is highly variable across individual mice. Log2(TPM + 1) values of all circulating genes of the validation cohort. DSP: double spun plasma; SSP: single spun plasma; TSP: triple spun plasma.

Supplemental figure 10F

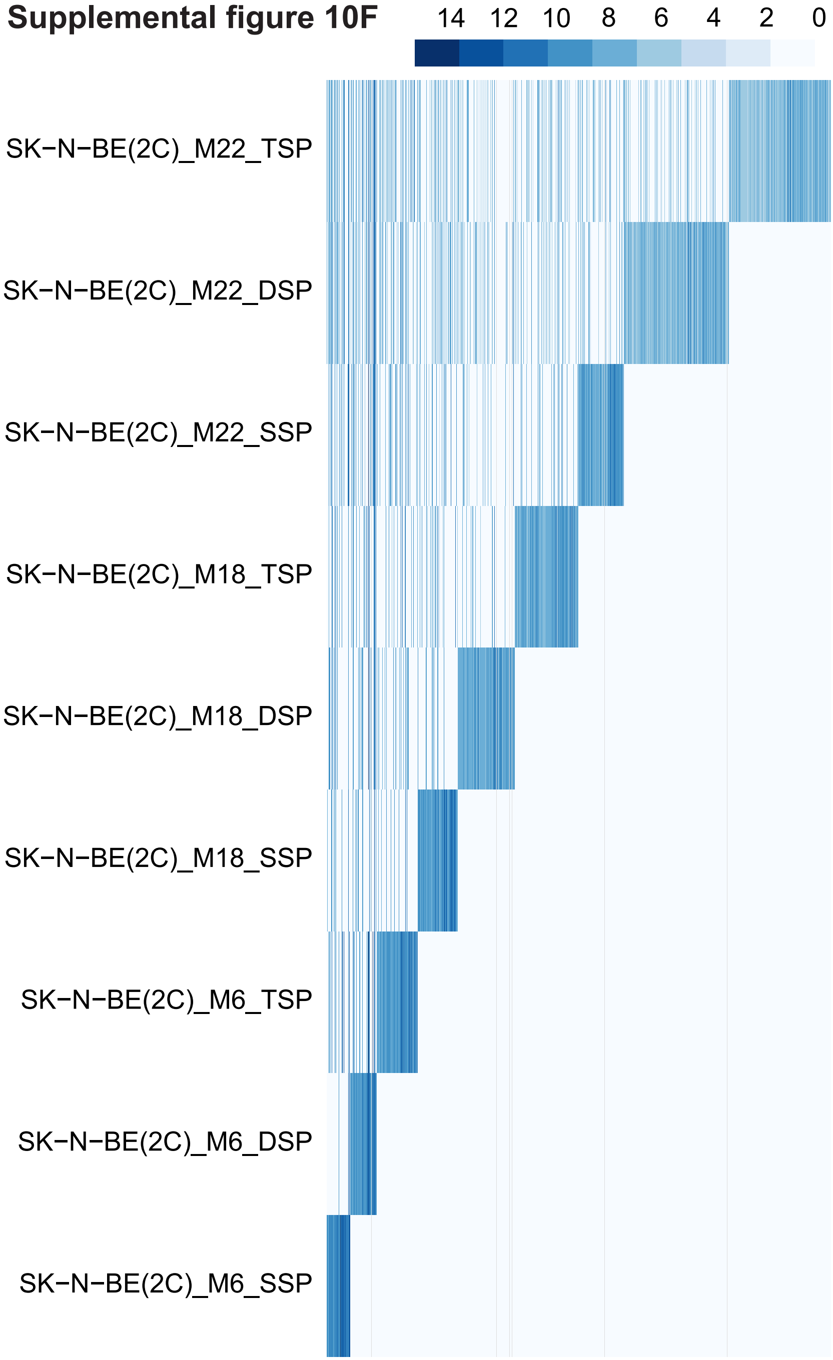

Supplemental figure 10A to F. The circulating tumour gene abundance profile is highly variable across individual mice. Log2(TPM + 1) values of all circulating genes of the validation cohort. DSP: double spun plasma; SSP: single spun plasma; TSP: triple spun plasma.

Supplemental figure 11

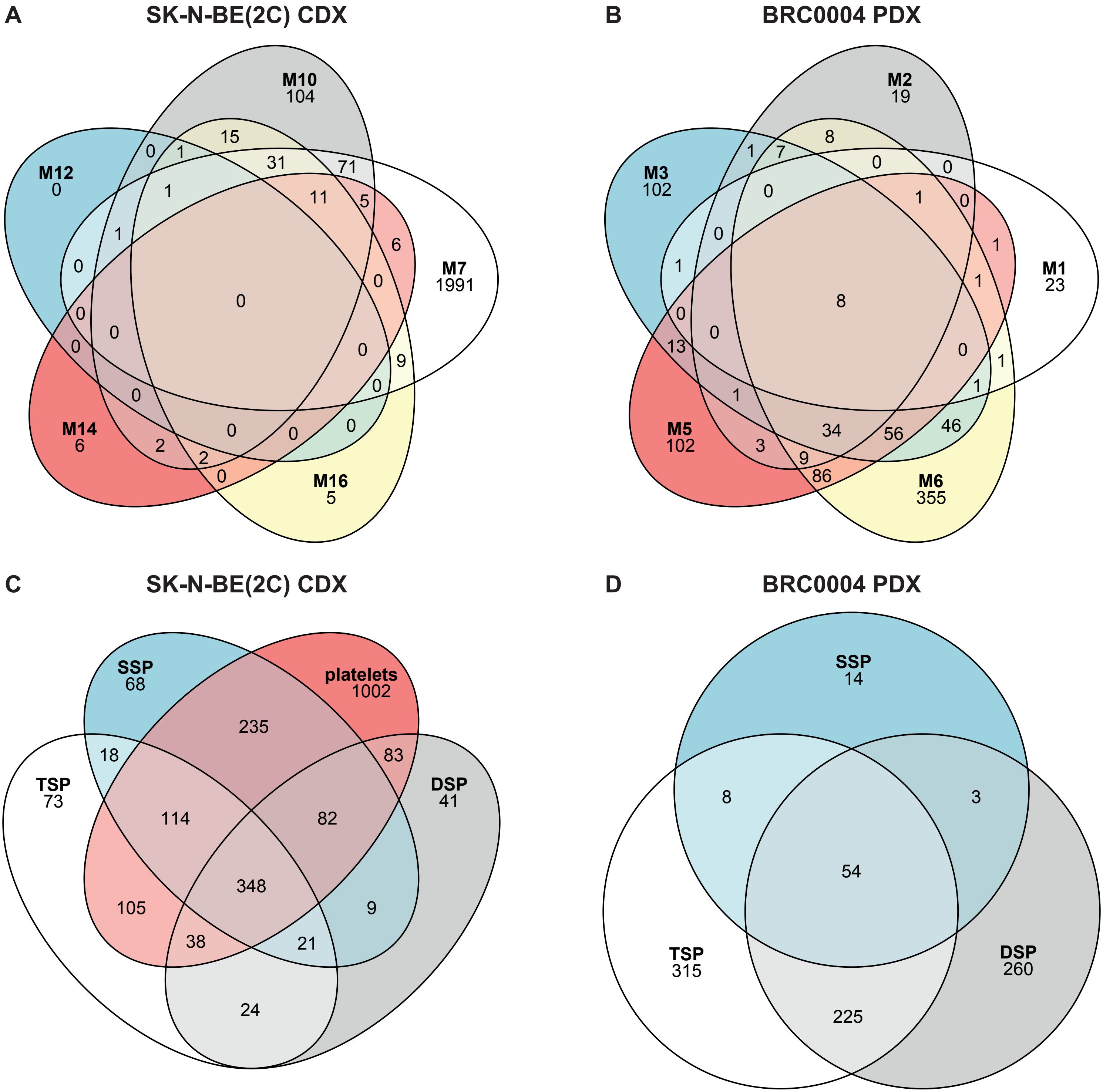

Supplemental figure 11. The circulating tumour gene profiles demonstrate common and distinct gene sets across different mice and across different liquid biopsy types. Overlap of the number of tumour-derived protein-coding genes (i.e. genes with ≥ 5 counts) across the different CDX (A) or PDX (B) mice (using all liquid biopsy types of the discovery cohort), as well as across the different liquid biopsy types for all CDX (C) or PDX (D) mice. DSP: double spun plasma; SSP: single spun plasma; TSP: triple spun plasma.

SUPPLEMENTAL TABLES (cf. Excel files)

Supplemental table 1. Overall, the SK-N-BE(2C) CDX and BRC0004 PDX sequencing data are of good quality. A. Quality control for the SK-N-BE(2C) CDX mice experiment. B. Quality control for the BRC0004 PDX mice experiment. For each RNA sample (RNA ID), the matching mouse ID, biomaterial ID and sample type is shown, as well as haemolysis levels measured by NanoDrop technology (absorbance of light at 414 nm) and RNA sequencing QC results. Sequencing depth: total read number; % trimmed: percentage total base pairs trimmed; % dups R1 or R2: percentage of downsampled R1 or R2 reads marked as duplicate; % GC R1 or R2: average GC content percentage of downsampled R1 or R2 reads; length R1 or R2: average sequence length (in bp) of downsampled R1 or R2 reads; % aligned to combined, human or mouse genome:percentage of downsampled reads aligned to the combined, human or mouse genome; exonic depth of combined, human or mouse genome: number of downsampled reads mapping to exons in the combined, human or mouse genome. The Sequin/ERCC ratio reflects RNA purification efficiency and should be relatively constant within a single experiment. DSP: double spun plasma; NA: not applicable; SSP: single spun plasma; TSP: triple spun plasma.

Supplemental table 2. The sequencing data of the validation cohort are of good quality. For each RNA sample (RNA ID), the matching mouse ID, biomaterial ID and sample type is shown, as well as haemolysis levels measured by NanoDrop technology (absorbance of light at 414 nm) and RNA sequencing QC results. Sequencing depth: total read number; % trimmed: percentage of total base pairs trimmed; % dups R1 or R2: percentage of downsampled R1 or R2 reads marked as duplicate; % GC R1 or R2: average GC content percentage of downsampled R1 or R2 reads; length R1 or R2: average sequence length (in bp) of downsampled R1 or R2 reads; % aligned to combined, human or mouse genome:percentage of downsampled reads aligned to the combined, human or mouse genome; exonic depth of combined, human or mouse genome: number of downsampled reads mapping to exons in the combined, human or mouse genome. The Sequin/ERCC ratio reflects RNA purification efficiency and should be relatively constant within a single experiment. DSP: double spun plasma; NA: not applicable; SSP: single spun plasma; TSP: triple spun plasma.

Supplemental table 3. Platelet content decreases upon successive centrifugation steps. Shown are the platelet counts per µl of plasma in single-spun (SSP), double-spun (DSP) and triple-spun (TSP) plasma.

Supplemental table 4. Endogenous RNA concentrations are calculated using Sequin spike-in controls. The concentration of Sequin spike-in controls added to each sample, is calculated based on the concentration (in attomol/µl) and length (in nucleotides) of each individual Sequin spike-in control.

Supplemental table 5. Gene set enrichment analyses on differentially abundant host genes between SSP, DSP and TSP demonstrate that murine platelet genes are enriched in SSP. For each xenograft model, the normalized enrichment score (NES), nominal P-value (nom P-value < 0.05), and false discovery rate q-value (FDR q-value <0.05) is indicated.
